# MPT0E028, a pan-HDAC inhibitor, ameliorates bleomycin-induced pulmonary fibrosis by promoting AT2-to-AT1 differentiation through the ATM/AMPK/FoxO1 pathway

**DOI:** 10.1186/s12929-026-01281-8

**Published:** 2026-07-21

**Authors:** Chia-Hao Liu, Hong-Sheng Lee, Jing-Ping Liou, Wun-Hao Cheng, Hung-Sheng Hua, Bing-Chang Chen, Chien-Huang Lin

**Affiliations:** 1 Graduate Institute of Medical Sciences, College of Medicine, Taipei Medical University, 250 Wu-Hsing Street, Taipei, 110 Taiwan; 2 Chen Wei-Tien Research Center of Thoracic Medicine, Taipei Medical University, Taipei, Taiwan; 3 School of Pharmacy, College of Pharmacy, Taipei Medical University, Taipei, Taiwan; 4 School of Respiratory Therapy, College of Medicine, Taipei Medical University, 250 Wu-Hsing Street, Taipei, 110 Taiwan; 5 Respiratory Therapy, Division of Pulmonary Medicine, Department of Internal Medicine, Wan Fang Hospital, Taipei Medical University, Taipei, Taiwan

**Keywords:** MPT0E028, T1α, Ataxia-telangiectasia mutated (ATM), Type 2 alveolar epithelial (AT2) cells, Pulmonary fibrosis

## Abstract

**Background:**

Persistent injury and impaired regeneration of the alveolar epithelium are key contributors to the pathogenesis of pulmonary fibrosis. In idiopathic pulmonary fibrosis (IPF), type 2 alveolar epithelial (AT2) cells fail to fully differentiate into type 1 alveolar epithelial (AT1) cells, remaining instead in a transitional state. Histone deacetylase (HDAC) inhibitors are promising therapeutic agents for pulmonary fibrosis. Therefore, this study investigated whether MPT0E028, a pan-HDAC inhibitor, ameliorated bleomycin (BLM)-induced pulmonary fibrosis in a therapeutic model of mice by promoting AT2-to-AT1 cell differentiation.

**Methods:**

The effects of MPT0E028 on pulmonary fibrosis were assessed by evaluating the expression of fibrogenic proteins and cell markers of AT1 (T1α and aquaporin 5 [AQP5]), AT2 (surfactant protein C [SPC]) and alveolar epithelial transitional cells (Keratin 8 [KRT8]) in a therapeutic model of BLM-induced pulmonary fibrosis in mice. The role of the ataxia-telangiectasia mutated (ATM)/AMP-activated protein kinase (AMPK)/forkhead box O1 (FoxO1) signaling pathway in MPT0E028-induced T1α expression was examined in murine AT2 cells (MLE-12 cells).

**Results:**

Administration of MPT0E028 significantly reduced fibrosis scores; suppressed the expression of connective tissue growth factor, collagen I, fibronectin, and α-smooth muscle actin; and improved lung function in the therapeutic model of BLM-induced pulmonary fibrosis in mice. MPT0E028 enhanced the expression of T1α and AQP5 but reduced the expression of SPC and KRT8 in lung tissues from BLM-treated mice. In MLE-12 cells and primary human AT2 cells, MPT0E028 upregulated T1α and AQP5 expression in a time-dependent manner, with this accompanied by a decrease in SPC expression. AS1842856, an FoxO1 inhibitor, and FoxO1 siRNA transfection inhibited MPT0E028-stimulated T1α expression, whereas transfection with FoxO3 siRNA had no effect. FoxO1 siRNA transfection also inhibited MPT0E028-stimulated T1α-luciferase activity. MPT0E028 induced FoxO1 serine phosphorylation, increased FoxO1 recruitment to the T1α promoter, and enhanced FoxO1-luciferase activity. Compound C, an AMPK inhibitor, and AMPK siRNA transfection suppressed MPT0E028-stimulated T1α expression, and compound C also inhibited MPT0E028-promoted FoxO1 recruitment to the T1α promoter. MPT0E028 induced AMPK phosphorylation in a time-dependent manner and increased ATM acetylation and phosphorylation in MLE-12 cells. ATM siRNA transfection suppressed MPT0E028-induced T1α expression, AMPK and FoxO1 serine phosphorylation, FoxO1 recruitment to the T1α promoter, and FoxO1-luciferase activity. MPT0E028 induced FoxO1 phosphorylation in AT2 cells in the therapeutic model of BLM-induced pulmonary fibrosis in mice.

**Conclusions:**

MPT0E028 is the first pan-HDAC inhibitor shown to activate ATM acetylation-mediated AMPK/FoxO1 signaling to induce AT2-to-AT1 differentiation in a therapeutic model of BLM-induced pulmonary fibrosis in mice. Administration of MPT0E028 after BLM challenge effectively ameliorated pulmonary fibrosis by suppressing fibrogenic protein expression and promoting AT2-to-AT1 cell differentiation. These results suggest that MPT0E028 holds potential as a therapeutic agent for IPF treatment.

**Supplementary Information:**

The online version contains supplementary material available at 10.1186/s12929-026-01281-8.

## Background

Idiopathic pulmonary fibrosis (IPF) is a devastating lung disease characterized by a poor prognosis and high mortality rate [1]. Although current antifibrotic treatments, such as pirfenidone and nintedanib, can slow disease progression, they neither restore impaired lung function nor serve as a cure. Moreover, both treatments are associated with substantial adverse drug reactions [2, 3]. In consideration of these factors, novel therapeutic strategies must be identified. Pulmonary fibrosis results from recurrent alveolar injury caused by factors such as genetic predisposition, environmental exposures, and infections. These injuries trigger the activation of myofibroblasts expressing α-smooth muscle actin (α-SMA), leading to excessive accumulation of extracellular matrix (ECM) components, such as fibronectin and collagen [4]. Therefore, an effective therapeutic approach for IPF should promote lung resolution, which involves the clearance of fibroproliferative stimuli, elimination of activated myofibroblasts, degradation of ECM, and re-epithelialization of damaged lung epithelium and alveoli [5].

Recurrent injury and impaired regeneration of the alveolar epithelium are key drivers of pulmonary fibrosis [6, 7]. The alveolar epithelium is composed of type 1 alveolar epithelial (AT1) cells and type 2 alveolar epithelial (AT2) cells. AT1 cells, which specialize in gas exchange, are characterized by the expression of specific markers, such as aquaporin 5 (AQP5) and T1α. By contrast, AT2 cells secrete surfactant proteins, including surfactant protein C (SPC), which is uniquely expressed by AT2 cells and plays a critical role in reducing surface tension in the alveoli, thereby preventing airspace collapse [8]. Additionally, AT2 cells function as progenitor cells, with self-renewal ability and the ability to differentiation into AT1 cells to facilitate alveolar epithelial regeneration following injury [7, 8]. Single-cell RNA sequencing analyses have revealed impaired alveolar epithelial cell function as a key contributor to IPF pathogenesis [9]. In patients with IPF, AT2 cells remain in a transitional state rather than effectively differentiating into AT1 cells [9]. A study demonstrated that deletion of the cell division control protein 42 homolog impairs AT2-to-AT1 differentiation, leading to increased mechanical tension in lung tissue and activation of the transforming growth factor-beta signaling pathway. This disruption promoted the progression of pulmonary fibrosis in aged mice and in mice subjected to lung lobe resection [10]. Collectively, these findings suggest that promoting AT2-to-AT1 differentiation may be a therapeutic strategy for resolving pulmonary fibrosis.

MPT0E028 [(3–1-benzenesulfonyl-2,3-dihydro-1H-indol-5-yl)-N-hydroxy-acrylamide], a pan-histone deacetylase (HDAC) inhibitor, was revealed to inhibit Akt signaling, thereby inducing apoptosis in B-cell lymphoma cells [11]. Additionally, MPT0E028 exhibited anticancer efficacy against human colorectal cancer HCT116 cells by inducing apoptosis both in vitro and in vivo [12]. Furthermore, a study by our group revealed that MPT0E028 enhances mitogen-activated protein kinase phosphatase-1 (MKP-1) acetylation and activation, leading to the downregulation of connective tissue growth factor (CTGF) expression in human lung fibroblasts and prevention of bleomycin (BLM)-induced pulmonary fibrosis [13]. A growing body of evidence supports the potential of HDAC inhibitors as therapeutic agents for pulmonary fibrosis [14, 15]. For example, suberoylanilide hydroxamic acid (SAHA) was demonstrated to induce apoptosis in IPF myofibroblasts by downregulating the antiapoptotic gene Bcl-xL and upregulating the proapoptotic gene *Bak*. Moreover, post-treatment with SAHA exhibited therapeutic efficacy in alleviating pulmonary fibrosis in a BLM-induced mouse model [16]. Moreover, a study indicated that SAHA promotes the interaction of GATA binding protein 6 with specificity protein 1 and p300, thereby enhancing AQP5 expression in murine AT2 cells (MLE-15 cells) [17]. These findings suggest that HDAC inhibitors may facilitate the differentiation of AT2 into AT1 cells during pulmonary fibrosis. However, whether MPT0E028 promotes AT2-to-AT1 differentiation to facilitate the resolution of pulmonary fibrosis remains unknown.

Ataxia-telangiectasia mutated (ATM) is a serine/threonine kinase implicated in ataxia-telangiectasia, a rare autosomal recessive neurodegenerative disorder [18]. Acetylation of ATM was identified as an essential factor for promoting ATM autophosphorylation at serine 1981 and promoting activation [19]. Trichostatin A (TSA), the pan-HDAC inhibitor, was demonstrated to promote the acetylation of ATM, thereby increasing ATM phosphorylation at serine 1981 and activating ATM-dependent DNA damage signaling pathways [20]. A study revealed that ATM-deficient mice exhibit increased numbers of neutrophils and lymphocytes, elevated levels of proinflammatory cytokines (e.g., interleukin 6 and tumor necrosis factor α), and obvious bronchial epithelial disruption in hydrogen chloride–induced acute lung inflammation [21]. Moreover, deletion of ATM exacerbates BLM-induced pulmonary fibrosis in mice and reduces AT2 cell viability [22]. ATM was also implicated in insulin-like growth factor-1-induced AMPK phosphorylation in human pancreatic cancer cells (PANC-1 cells) [23]. AMPK activation is known to enhance energy metabolism, thereby promoting AT2-to-AT1 differentiation and promoting alveolar recovery following injury [24]. Additionally, AMPK was reported to require forkhead box protein O1 (FoxO1) to alleviate cigarette smoke extract–induced endoplasmic reticulum stress and thereby protect human bronchial cells from apoptosis [25]. FoxO1 activation was revealed to increase the interaction of FoxO1 with the transcription factor Nkx2.1, leading to the downregulation of surfactant protein synthesis in AT2 cells and thus stimulating AT2-to-AT1 differentiation [26]. However, whether the ATM/AMPK/FoxO1 pathway is involved in MPT0E028-induced AT2-to-AT1 differentiation remains unclear.

In this study, we investigated whether MPT0E028 ameliorates BLM-induced pulmonary fibrosis in a therapeutic mouse model by activating ATM acetylation-mediated AMPK/FoxO1 signaling, thereby promoting AT2-to-AT1 cell differentiation. MPT0E028 was administered starting on day 10 following BLM treatment significantly ameliorated pulmonary fibrosis, reduced fibrogenic protein expression, and mitigated lung function decline. Moreover, MPT0E028 treatment promoted AT2-to-AT1 differentiation, as evidenced by changes in the expression of the AT2 cell marker SPC and AT1 cell markers T1α and AQP5, both in vivo and in vitro. Furthermore, MPT0E028 facilitated AT2-to-AT1 cell differentiation by promoting ATM acetylation, thereby activating the ATM/AMPK/FoxO1 signaling pathway in murine AT2 cells (MLE-12 cells). These findings support the therapeutic potential of MPT0E028 in pulmonary fibrosis.

## Materials and methods

### Materials

MPT0E028 [(3–1-benzenesulfonyl-2,3-dihydro-1H-indol-5-yl)-N-hydroxy-acrylamide] with a purity of 98% was synthesized by Dr. J. P. Liou (Taipei Medical University, Taipei, Taiwan). Primary antibodies specific for α-SMA, CTGF, fibronectin, SPC, ATM, horseradish peroxidase (HRP)-conjugated anti-Syrian hamster immunoglobulin G (IgG), and Alexa Fluor 488-conjugated anti-Syrian hamster IgG were purchased from Abcam (Cambridge, UK). The antibody specific for AQP5 was purchased from Genetex (Irvine, CA, USA). Antibodies specific for acetylated lysine, collagen I, phosphorylated (p)-AMPK at Thr172, AMPK, FoxO1, and FoxO3 were obtained from Cell Signaling Technology (Danvers, MA, USA). In addition, 4-(2-Hydroxyethyl)−1-piperazineethanesulfonic acid (HEPES), anti-α-tubulin antibody, anti-phospho-serine (anti-p-Ser) antibody, β-estradiol, hydrocortisone, insulin, sodium selenite, transferrin, AS1842856, KU-60019, compound C, protein A mag beads, scrambled small interfering RNA (siRNA), AMPK siRNA, ATM siRNA, FoxO1 siRNA, FoxO3 siRNA, and the chromatin immunoprecipitation (ChIP) assay kit were obtained from Sigma-Aldrich (St. Louis, MO, USA). Immunoprecipitation (IP) lysis buffer, cocktail inhibitor, Dulbecco’s modified eagle medium/nutrient mixture F-12 (DMEM/F12) powder, fetal bovine serum (FBS), L-glutamine, antibiotic–antimycotic, 0.25% trypsin-ethylenediaminetetraacetic acid (EDTA), and anti-keratin 8 (KRT8) antibody were procured from ThermoFisher Scientific (Waltham, MA, USA). Lipofectamine 3000, antibodies for T1α, p-ATM at Ser1981, Alexa Fluor 555-conjugated anti-rabbit IgG, and Alexa Fluor 647-conjugated anti-rabbit IgG were procured from Invitrogen Life Technologies (Carlsbad, CA, USA). Growth factor reduced Matrigel was sourced from Corning Life Sciences (Corning, NY, USA). HRP-conjugated goat antirabbit IgG and HRP-conjugated goat antimouse IgG were acquired from Croyez Bioscience (Taipei, Taiwan). An immunohistochemistry (IHC) assay kit was purchased from Leica Biosystems (Wetzlar, Germany). A FoxO1 reporter kit was procured from BPS Bioscience (San Diego, CA, USA). Additionally, 2 × Tools Tag PCR MasterMix with loading dye and proteinase K were purchased from BIOTOOLS (Taipei, Taiwan), and 4′,6-Diamidino-2-phenylindole (DAPI) Fluoromount-G was procured from SouthernBiotech (Birmingham, AL, USA).

### Cell culture

The mouse lung AT2 epithelial cell line (MLE-12) was purchased from the American Type Culture Collection (Manassas, VA, USA). The MLE-12 cells were maintained in DMEM/F12 supplemented with 2% FBS, 0.005 mg/mL insulin, 0.01 mg/mL transferrin, 30 nM sodium selenite, 10 nM hydrocortisone, 10 nM β-estradiol, 2 mM additional L-glutamine, and 10 mM HEPES. Cell cultures were incubated in a humidified incubator at 37 °C with 5% CO_2_. The cells used in the experiments were between passages 5 and 15. Upon reaching approximately 90% confluence in 75 T flasks, the MLE-12 cells were subcultured in 60-mm dishes for Western blotting analysis, IP, and ChIP or in 12-well culture plates for immunofluorescence (IF) assays. The primary human lung AT2 epithelial cell and Complete Culture Medium were purchased from the iCell Bioscience Inc. (Shanghai, China). The primary human lung AT2 epithelial cells were maintained in Complete Culture Medium supplemented with 10% FBS. Cell cultures were incubated in a humidified incubator at 37 °C with 5% CO_2_. The cells used in the experiments were between passages 3 and 10. Upon reaching approximately 90% confluence in 75 T flasks, the primary human lung AT2 epithelial cells were subcultured in 60-mm dishes for Western blotting analysis.

### Western blotting

In the in vitro experiments, MLE-12 cells were seeded in 60-mm dishes at a density of 2 × 10^5^ cells per dish. Once the cells had adhered, they were subjected to siRNA transfection, treatment with compound C (AMPK inhibitor, 1–10 μM), AS1842856 (FoxO1 inhibitor, 0.3–3 μM) or KU-60019 (ATM inhibitor, 0.3–3 μM), followed by MPT0E028 (0.3 μM) treatment at the indicated time intervals. In the in vivo experiments, whole lung tissues were collected from designated animal studies. The lung tissue samples and cultured cells were both lysed using a lysis buffer supplemented with cocktail inhibitors. The total protein concentration was quantified using the bicinchoninic acid assay. Protein lysates were separated through sodium dodecyl sulfate–polyacrylamide gel electrophoresis and subsequently transferred into polyvinylidene difluoride membranes. The membranes were blocked with 5% nonfat milk for 1 h at room temperature and then incubated overnight at 4 °C with primary antibodies specific to collagen I, fibronectin, α-SMA, CTGF, T1α, AQP5, SPC, p-AMPK (Thr172), AMPK, phosphorylated serine (p-Ser), acetylated lysine, p-ATM (Ser1981), ATM, FoxO1, FoxO3, or α-tubulin. Following primary antibody incubation, the membranes were exposed to HRP-conjugated secondary antibodies for 1 h at room temperature. Protein bands were visualized using electrochemiluminescence reagents and imaged using the ChemiDoc MP imaging system (Bio-Rad; Hercules, CA, USA). Band intensities were quantified using Image-Pro analysis software (Media Cybernetics; Rockville, MD, USA).

### Immunofluorescence (IF) staining

The MLE-12 cells were cultured on coverslips precoated with 2.5% v/v Matrigel in a 12-well plate at a density of 2.5 × 10^4^ cells per well. After adherence, the cells were treated with MPT0E028 (0.3 μM) for 24 h. Following treatment, the cells were fixed with 4% paraformaldehyde for 10 min at room temperature. The fixed coverslips were blocked with 0.5% w/w bovine serum albumin in phosphate-buffered saline (PBS) for 1 h. Subsequently, the cells were incubated with primary antibodies against T1α and SPC overnight at 4 °C. Secondary antibodies conjugated with Alexa Fluor 488, and Alexa Fluor 647 were used to stain the hamster and rabbit primary antibodies for another 1 h. Nuclei were counterstained using a mounting medium containing DAPI.

The collected lung tissues were carefully infused with 10% formaldehyde via the trachea and were subsequently submerged in a sample vial containing 10 mL of 10% formaldehyde for a minimum of 48 h to ensure complete fixation. The tissue sections were treated again with xylene, alcohol (100%, 95%, 75%, and 60%), and ddH_2_O to remove residual paraffin. Subsequently, the deparaffinized sections were incubated with primary antibodies against FoxO1, p-Ser and SPC overnight at 4 °C. Secondary antibodies conjugated with Alexa Fluor 488, Alexa Fluor 555 and Alexa Fluor 647 were used to stain the primary antibodies for another 1 h. Nuclei were counterstained using a mounting medium containing DAPI. IF images were acquired using a fluorescence microscope (Echo Revolve; San Diego, CA, USA).

### Small interfering RNA (siRNA) transfection

The MLE-12 cells were seeded in 60-mm dishes at a density of 1 × 10^5^ cells per dish. After the cells were allowed to adhere, transfection was performed using Lipofectamine 3000 containing scrambled siRNA, ATM siRNA, AMPK siRNA, FoxO1 siRNA, and FoxO3 siRNA. After 24 h of siRNA transfection, the cells were treated with MPT0E028 (0.3 μM) for the indicated time intervals. Following treatment, protein expression levels were assessed through Western blotting.

### Luciferase reporter assay

The MLE-12 cells were seeded in 12-well plates at a density of 5 × 10^4^ cells per well. After cell attachment, the cells were transfected with 0.3 μg FoxO1-Luc or 0.3 μg T1α-Luc and 0.1 μg Lac Z plasmids by using Lipofectamine 3000. Following 24 h of transfection, the cells were treated with MPT0E028 (0.01, 0.03, 0.1, and 0.3 μM) for an additional 24 h. Luciferase activity was measured using the Luciferase assay system (E1500, Promega, Madison, WI, USA), and the results were normalized to the expression levels of LacZ.

### Immunoprecipitation (IP)

The MLE-12 cells were seeded in 60-mm dishes at a density of 2 × 10^5^ cells per dish. After cell attachment, the cells were treated with MPT0E028 (0.3 μM) at the specified time intervals. Following treatment, the cells were harvested and lysed in IP lysis buffer supplemented with cocktail inhibitors. The lysates were then centrifuged at 6,000 × g for 10 min at 4 °C. The resulting supernatants were incubated overnight at 4 °C with an anti-ATM antibody or anti-FoxO1 antibody and protein A beads. The immunoprecipitated samples were washed three times with IP lysis buffer to remove nontarget proteins and were subsequently analyzed through Western blotting.

### Chromatin immunoprecipitation (ChIP) assay

The MLE-12 cells were seeded in 60-mm dishes at a density of 2 × 10^5^ cells per dish. After cell attachment, the cells were transfected with ATM siRNA or treated with compound C (AMPK inhibitor, 10 μM), followed by MPT0E028 (0.3 μM) treatment at the indicated time intervals. Subsequently, the cells were fixed with 10% formaldehyde for 10 min. The reaction was quenched through the addition of 0.1 M glycine solution for 25 min. The cells were subsequently lysed, and the cell lysates were sonicated and centrifuged at 14,000 × g at 4 °C for 10 min. Each sample containing protein A beads was incubated with a FoxO1-specific antibody or rabbit IgG overnight at 4 °C. Following incubation, DNA was purified using the ChIP assay kit in accordance with the manufacturer’s instructions. The FoxO1 binding site on the T1α promoter region was amplified through polymerase chain reaction under the following conditions: 45 cycles of denaturation at 95 °C for 30 s, annealing at 50 °C for 30 s, and extension at 72 °C for 30 s. The following primer sequences were used for amplification: 5′-ATC CAT CCA CGT GCC TTG-3′ (sense) and 5′-TTG TTC CAC TAC AGG AGA-3′ (antisense).

### Therapeutic model of bleomycin (BLM)-induced pulmonary fibrosis in mice

All animal study protocols were approved by the Institutional Animal Care and Use Committee of Taipei Medical University (LAC-2020–0170). Eight-week-old C57BL/6 mice were procured from the National Laboratory Animal Center (Taipei, Taiwan) and randomly assigned to seven experimental groups: (1) sham + vehicle control, *n* = 9, (2) BLM + vehicle control, *n* = 9, (3) BLM + MPT0E028 (25 mg/kg/day), *n* = 10, (4) BLM + MPT0E028 (50 mg/kg/day),* n* = 12, (5) BLM + MPT0E028 (100 mg/kg/day), *n* = 10, (6) BLM + pirfenidone (200 mg/kg/day), *n* = 10 and (7) BLM + nintedanib (60 mg/kg/day),* n* = 10. The mice were anesthetized with 1%–5% isoflurane and subjected to intratracheal administration of BLM (0.3 U/kg). The sham group received an equivalent volume of PBS injection. MPT0E028, pirfenidone, and nintedanib were dissolved in a carboxymethylcellulose (CMC) solution composed of 0.5 wt% CMC, 0.1 wt% Tween 80, and 5 wt% dextran in ddH_2_O. Oral administration of MPT0E028 (25–100 mg/kg/day), pirfenidone (200 mg/kg/day), and nintedanib (60 mg/kg/day) was initiated on day 10 after BLM challenge and was continued until day 31. On day 32, all mice were sacrificed, and their lung tissues were harvested for subsequent analyses.

### Histology and immunohistochemistry (IHC)

The collected lung tissues were carefully infused with 10% formaldehyde via the trachea and were subsequently submerged in a sample vial containing 10 mL of 10% formaldehyde for a minimum of 48 h to ensure complete fixation. The fixed lung tissues were then embedded in paraffin. Xylene, alcohol (100%, 95%, 75%, and 60%), and ddH_2_O were subsequently used to detach the paraffin from the tissue sections. The tissue sections were treated again with xylene, alcohol (100%, 95%, 75%, and 60%), and ddH_2_O to remove residual paraffin. The deparaffinized sections were subjected to hematoxylin and eosin (H&E) staining to enable examination of the morphology and structure of the lung tissues. For IHC staining, antigen retrieval was performed by heating the sections to 95 °C in EDTA buffer (pH 9.0) for 1 h. An IHC assay kit was used to detect the expression of fibronectin, α-SMA, CTGF, AQP5, T1α, SPC and KRT8. Additionally, Masson’s trichrome staining was performed to visualize the collagen distribution in the lung tissues. All histological images were analyzed using a MoticEasyScan Pro 6 (Motic; HK, China).

### Lung function test

To evaluate pulmonary function, the mice used in this study were anesthetized with 4% isoflurane and subsequently maintained under 1%–2% isoflurane. A tracheotomy was performed to establish a secure airway connection to a ventilator system (Flexivent, SCIREQ; MTL, QC, Canada) for pulmonary function measurements. To minimize diaphragm interruption, pancuronium (2.5 mg/kg) was administered intraperitoneally prior to measurement. Once the mice achieved a stable respiratory rate, lung function tests were conducted under a tidal volume of 10 mL/kg, a positive end-expiratory pressure of 3 cmH_2_O, and a respiratory rate of 160 breaths per minute. Key pulmonary parameters, including lung compliance, lung elastance, airway resistance, and forced expiratory volume at 0.1 s (FEV_0.1_) were measured in accordance with the manufacturer’s instructions.

### Statistical analysis

All experimental data are presented as means ± standard errors of the mean (SEM). For graphical presentation, individual data points from independent experiments are shown as scatter plots with the median indicated by a horizontal line. The Shapiro–Wilk normality test was applied to assess data normality. For the animal studies, statistical comparisons were conducted using one-way analysis of variance (ANOVA) followed by Tukey’s post hoc test. For the time-dependent experiments, one-way ANOVA as performed with Dunnett’s post hoc test. For the siRNA knockdown and inhibitor studies, data were analyzed using one-way ANOVA followed by Tukey’s post hoc test. Statistical analyses were performed using GraphPad Prism 7 (GraphPad Software, San Diego, CA, USA). A *p* value of < 0.05 was considered significant in all analyses.

## Results

### MPT0E028 attenuated pulmonary fibrosis and fibrogenic protein expression in a therapeutic model of BLM-induced pulmonary fibrosis in mice

**Fig. 1:**
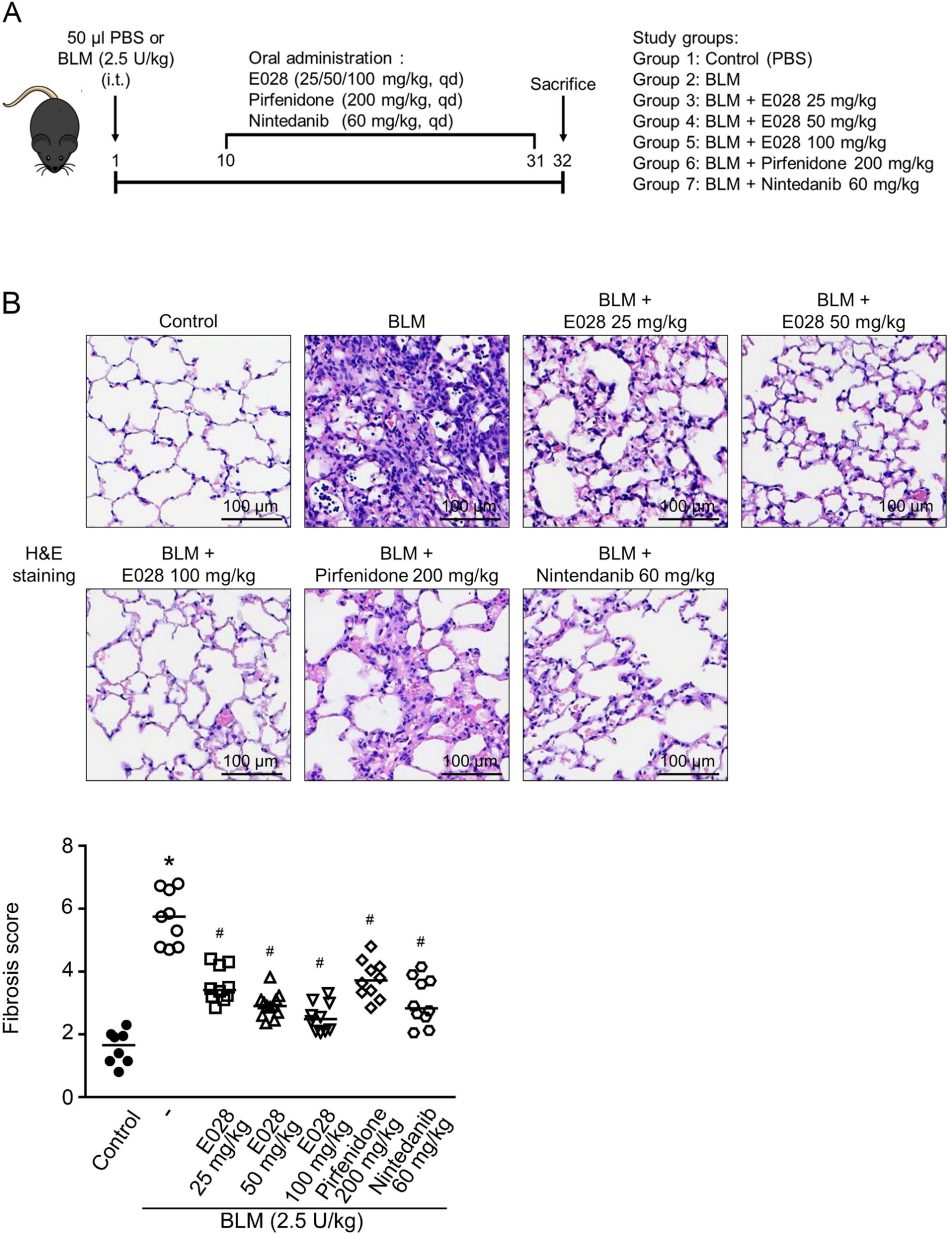
MPT0E028 ameliorated pulmonary fibrosis in a therapeutic model of BLM-induced pulmonary fibrosis in mice. **A** Schematic of the experimental design for the MPT0E028 intervention in an established pulmonary fibrosis model induced through BLM administration. Following intratracheal BLM administration, oral administration of MPT0E028 (25–100 mg/kg/day), pirfenidone (200 mg/kg/day), and nintedanib (60 mg/kg/day) was initiated once daily from day 10 to day 31. The mice were sacrificed on day 32 for further analysis. **B** After 32 days, whole lungs from mice were collected and fixed in formaldehyde for 24 h. The lungs were then embedded in paraffin and stained with H&E.; original magnification = 20 ×; scale bars = 100 μm. **C** Quantification of pulmonary fibrosis severity based on the modified Ashcroft scale. Fibrosis scores are presented as means ± SEMs. The n number has been specified in the Materials and methods section. *p < 0.05 compared with the sham + vehicle control group. ^#^*p* < 0.05 compared with the BLM + vehicle control group. *BLM* bleomycin, *E028* MPT0E028.

**Fig. 2:**
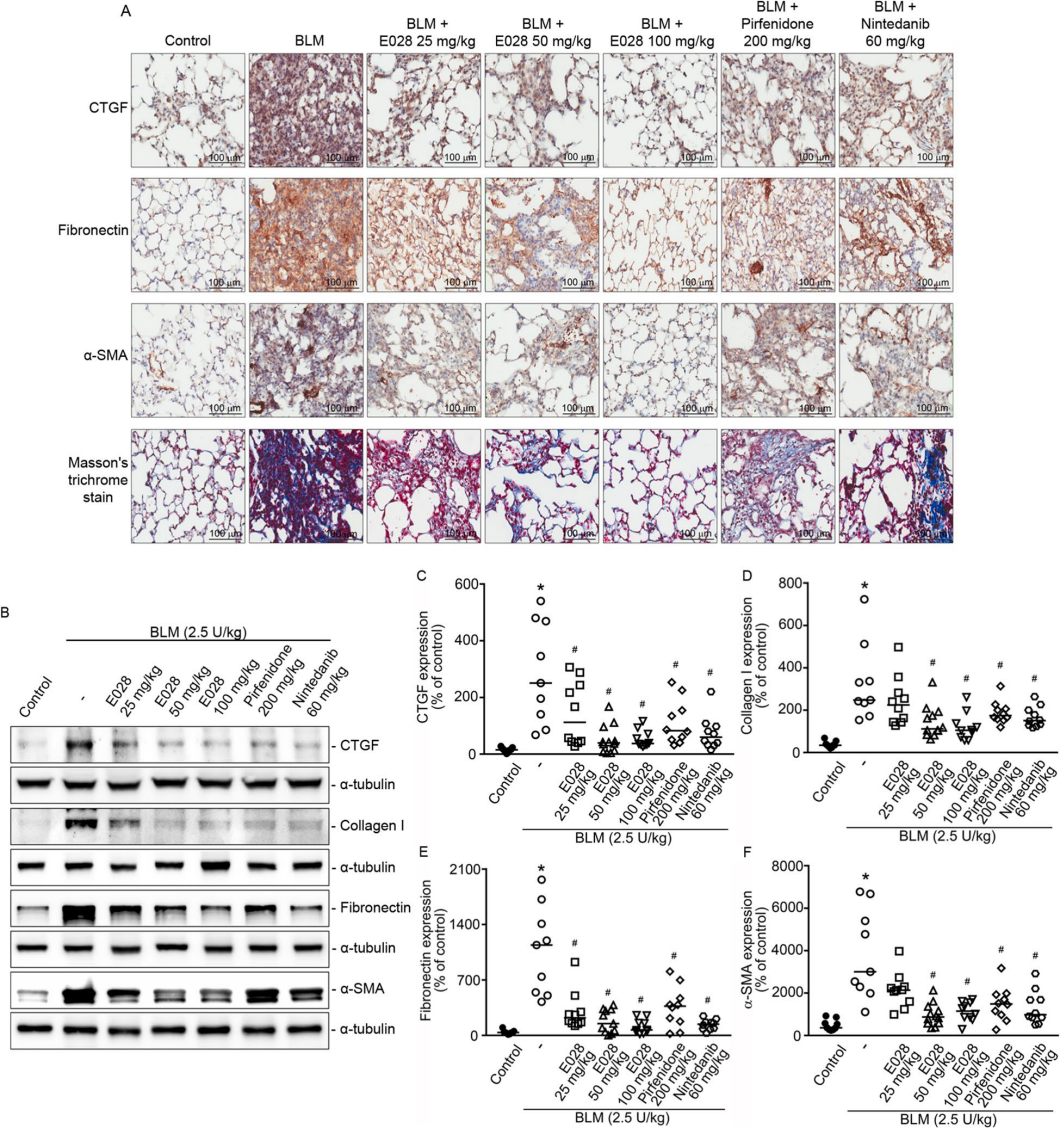
MPT0E028 suppressed the expression of fibrotic proteins in a therapeutic model of BLM-induced pulmonary fibrosis in mice. **A** Whole lungs were obtained from the mice and fixed with formaldehyde for 24 h, embedded in paraffin, and subjected to IHC staining for CTGF, fibronectin, and α-SMA and Masson’s trichrome staining for collagen. original magnification = 20 ×; scale bars = 100 μm. **B** After 32 days, whole lungs were collected from the mice and were lysed for immunoblotting to detect CTGF, collagen I, fibronectin, α-SMA, and α-tubulin. **C–F** Statistical analyses of the immunoblots for CTGF, collagen I, fibronectin, and α-SMA. The n number has been specified in the Materials and methods section. *p < 0.05 compared with the sham + vehicle control group. ^#^*p* < 0.05 compared with the BLM + vehicle control group. *BLM* bleomycin, *E028* MPT0E028

A previous study demonstrated that MPT0E028 pretreatment effectively prevents BLM-induced pulmonary fibrosis in mice [13]. In the present study, we investigated the therapeutic potential of MPT0E028 in a therapeutic model of BLM-induced pulmonary fibrosis in mice and conducted a comparative analysis with pirfenidone and nintedanib, two clinically approved treatments for IPF. We conducted a comparison of MPT0E028 with oral pirfenidone administered at 200 mg/kg and oral nintedanib administered at 60 mg/kg, with these concentrations selected on the basis of the highest dosages reported in the literature [27, 28]. MPT0E028 was administered orally at varying concentrations (25–100 mg/kg) to assess its dose-dependent effects. Figure 1A provides a schematic of the experimental design. MPT0E028 (25–100 mg/kg), pirfenidone (200 mg/kg), and nintedanib (60 mg/kg) were administered orally from day 10 following BLM treatment. H&E staining revealed increased pulmonary fibrosis in the BLM-treated group that did not receive medication, whereas treatment with MPT0E028 (25–100 mg/kg), pirfenidone (200 mg/kg), and nintedanib (60 mg/kg) effectively attenuated BLM-induced pulmonary fibrosis (Fig. 1B). The calculated fibrosis inhibition rates were as follows: 35.2% ± 6.4% for 25 mg/kg MPT0E028, 58.2% ± 4.5% for 50 mg/kg MPT0E028, 67.3% ± 7.6% for 100 mg/kg MPT0E028, 26.2% ± 11.6% for 200 mg/kg pirfenidone, and 57.0% ± 9.2% for 60 mg/kg nintedanib (Fig. 1C). The high dose (100 mg/kg) of MPT0E028 exhibited a comparable efficacy to that of pirfenidone (200 mg/kg) or nintedanib (60 mg/kg) in inhibiting BLM-induced pulmonary fibrosis. Furthermore, the expression of fibrogenic proteins, including CTGF, collagen, fibronectin, and α-SMA, was examined using histological staining and immunoblotting. As illustrated in Fig. 2A, Masson’s trichrome and IHC staining indicated that CTGF, fibronectin, α-SMA, and collagen were elevated in the lung interstitium of BLM-treated mice; however, a marked reduction was noted following treatment with MPT0E028 (25–100 mg/kg), pirfenidone (200 mg/kg), and nintedanib (60 mg/kg; Fig. 2A). Additionally, a Western blot analysis of whole lung lysates confirmed that the BLM-induced upregulation of CTGF, collagen I, fibronectin, and α-SMA was significantly diminished by MPT0E028 (25–100 mg/kg), pirfenidone (200 mg/kg), and nintedanib (60 mg/kg) treatment (Fig. 2B–F). These findings suggest that MPT0E028 markedly attenuated the expression of CTGF, collagen I, fibronectin, and α-SMA and further ameliorated BLM-induced pulmonary fibrosis.

### MPT0E028 enhanced lung function in a therapeutic model of BLM-induced pulmonary fibrosis in mice

**Fig. 3:**
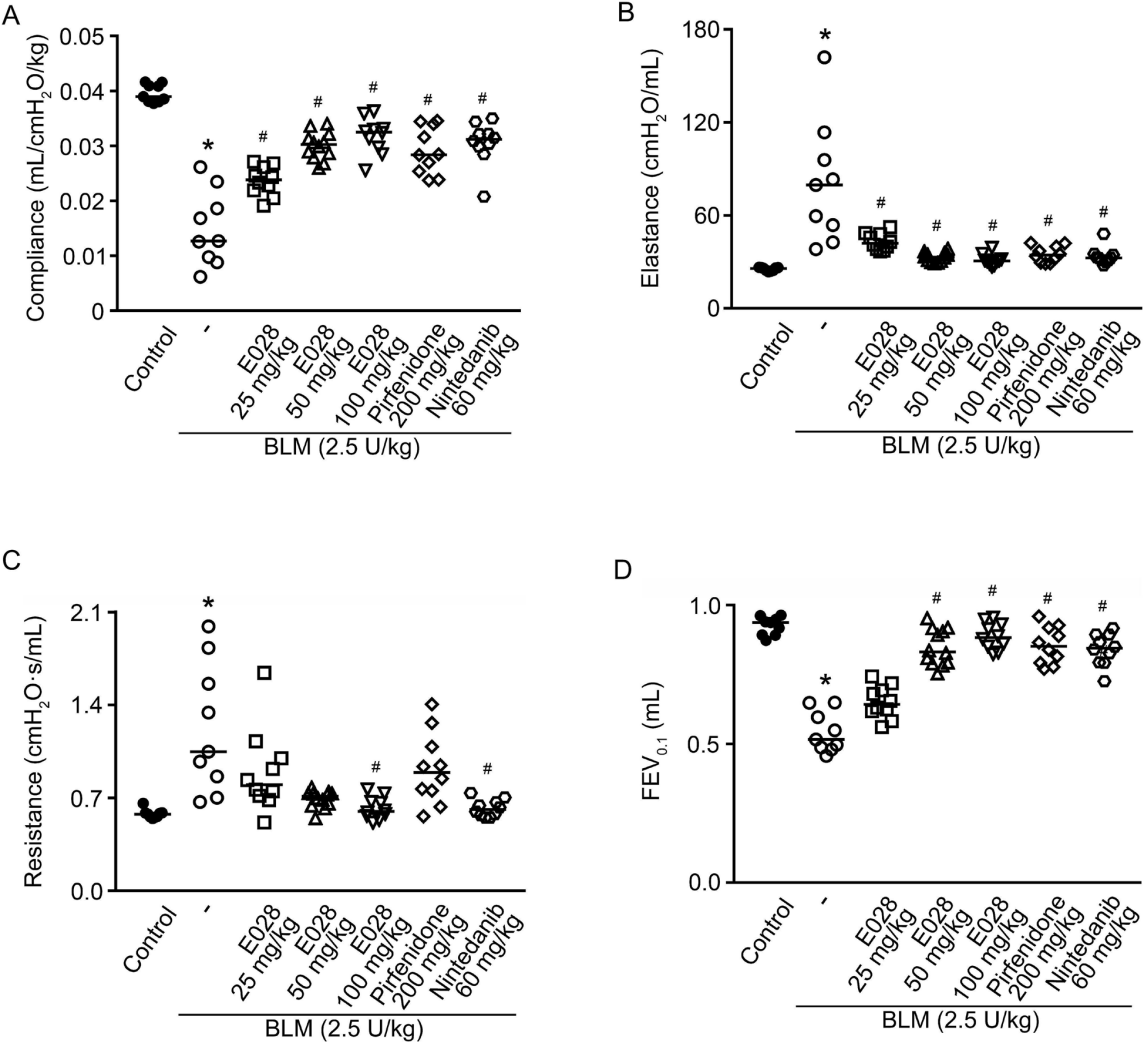
MPT0E028 attenuated lung function decline in a therapeutic model of BLM-induced pulmonary fibrosis in mice. On day 32, the mice were anesthetized with isoflurane and connected to a ventilator via tracheotomy to enable assessment of the **A** lung compliance, **B** lung elastance, **C** airway resistance, and **D** FEV_0.1_ under a tidal volume of 10 mL/kg, positive end-expiratory pressure of 3 cmH_2_O, and respiratory rate of 160 times/min. The n number has been specified in the Materials and methods section. All data are expressed as means ± SEMs. *p < 0.05 compared with the sham + vehicle control group. ^#^*p* < 0.05 compared with the BLM + vehicle control group. Abbreviations: BLM, bleomycin; E028, MPT0E028

To further investigate the therapeutic efficacy of MPT0E028 in BLM-induced pulmonary fibrosis, we performed lung function assessments to identify pulmonary physiological alterations. The BLM-treated group demonstrated impaired lung function with reduced lung compliance and FEV_0.1_ and increased lung elastance and airway resistance (Fig. 3A–D). Treatment with MPT0E028 (25–100 mg/kg), pirfenidone (200 mg/kg), and nintedanib (60 mg/kg) from day 10 following BLM treatment significantly reversed the BLM-induced reduction in lung compliance (Fig. 3A) and increase in elastance (Fig. 3B). Furthermore, MPT0E028 (25–100 mg/kg) and nintedanib (60 mg/kg) significantly reduced the BLM-induced increase in airway resistance, whereas pirfenidone (200 mg/kg) failed to produce a similar effect (Fig. 3C). Additionally, MPT0E028 (25–100 mg/kg), pirfenidone (200 mg/kg), and nintedanib (60 mg/kg) improved BLM-damaged FEV_0.1_ (Fig. 3D). These results suggest that MPT0E028 is comparable to pirfenidone and nintedanib in ameliorating pulmonary fibrosis and enhancing lung function.

### MPT0E028 promoted AT2-to-AT1 cell differentiation in a therapeutic model of BLM-induced pulmonary fibrosis, mouse AT2 cell line (MLE-12) and human primary AT2 cells

**Fig. 4:**
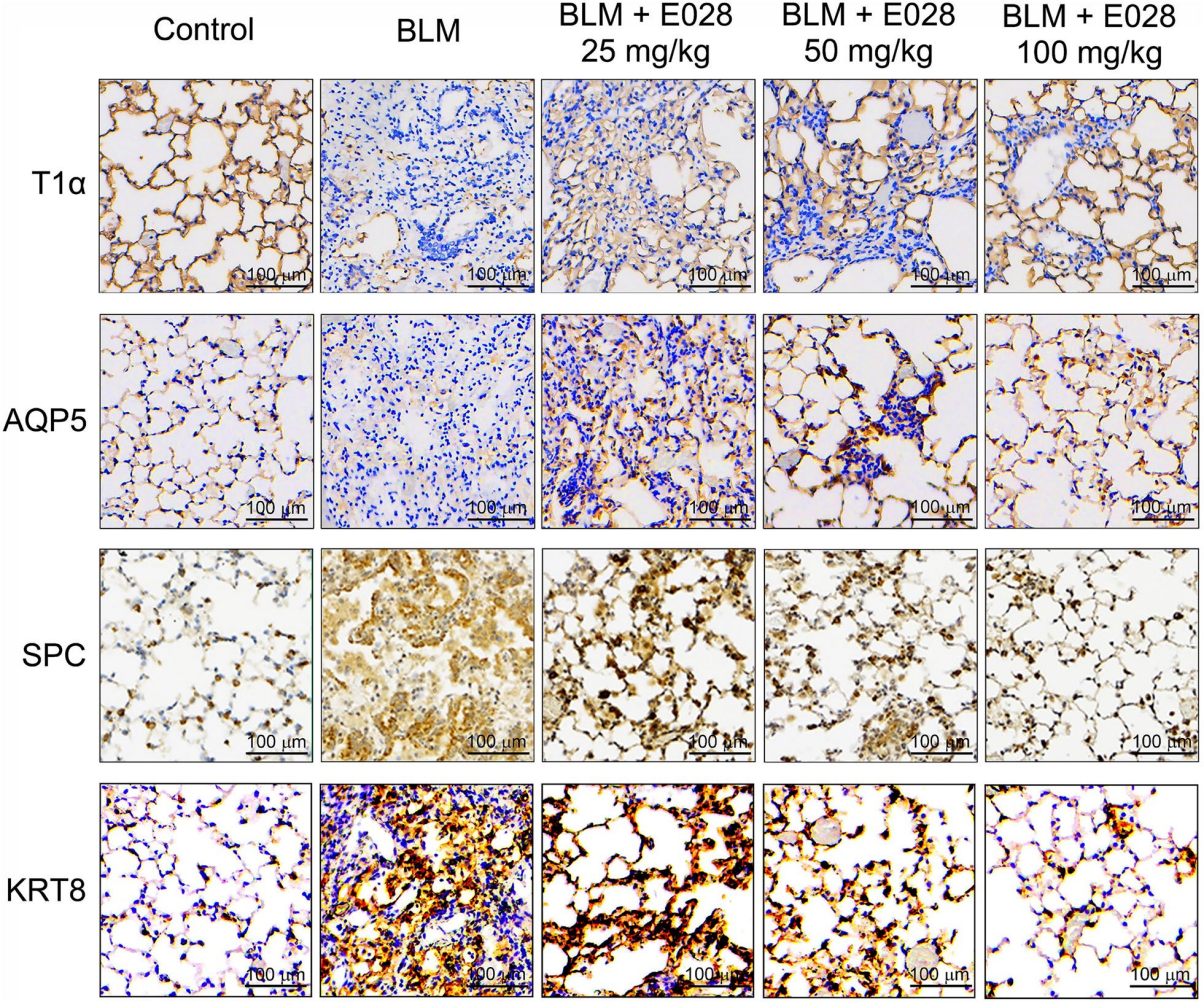
MPT0E028 increased T1α and AQP5 expression while reducing SPC and KRT8 expression in lung tissues from a therapeutic model of BLM-induced pulmonary fibrosis in mice. **A** On day 32, whole lungs were harvested from mice treated with PBS, BLM, or BLM + MPT0E028 (25, 50, and 100 mg/kg). Lung tissues were fixed in formaldehyde for 24 h, embedded in paraffin, and subjected to IHC staining for T1α, AQP5, SPC, and KRT8. The n number has been specified in the Materials and methods section. original magnification = 20 ×; scale bars = 100 μm. *AQP5* aquaporin 5, *BLM* bleomycin, *E028* MPT0E028, *FoxO1* forkhead box O1, *KRT8* Keratin 8, *SPC* surfactant protein C

**Fig. 5:**
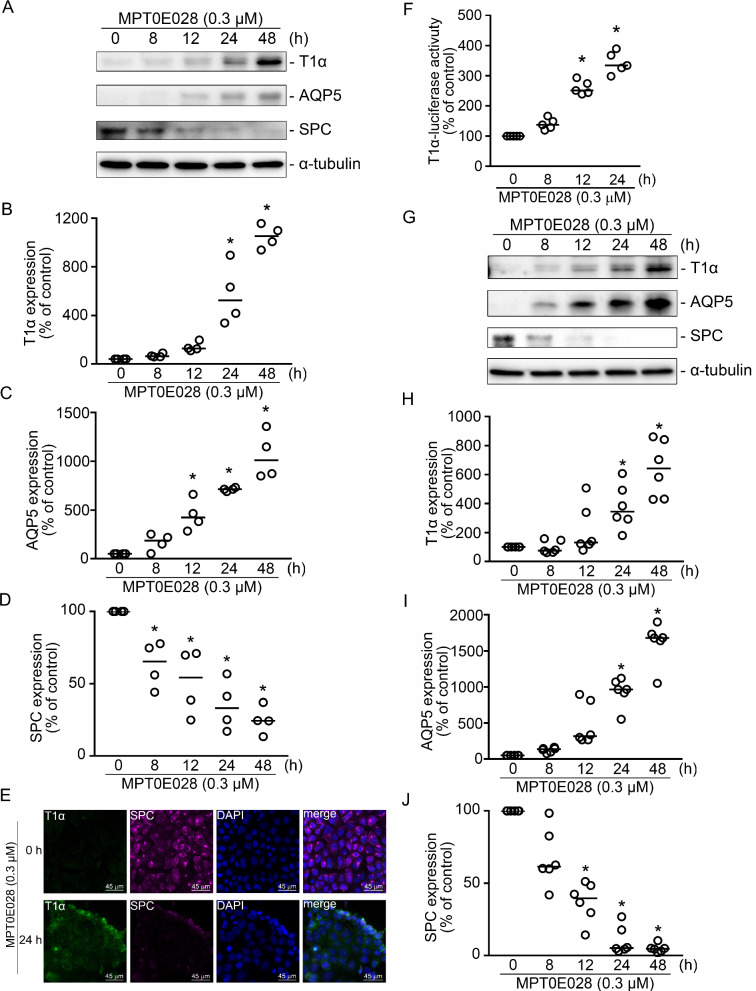
MPT0E028 upregulated T1α and AQP5 expression while suppressing SPC expression in mouse AT2 cell line (MLE-12 cells) and primary human AT2 cells. **A** MLE-12 cells were treated with 0.3 μM MPT0E028 for 8, 12, 24, and 48 h, followed by immunoblotting with antibodies specific for T1α, AQP5, SPC, and α-tubulin.** B**–**D** Quantitative analysis of T1α, AQP5, and SPC expression was conducted. Data are expressed as means ± SEMs from four independent experiments. *p < 0.05, compared with the non-treated group. **E** The cells were treated with 0.3 μM MPT0E028 for 24 h and fixed with 4% formaldehyde for 10 min. The fixed cells were stained for T1α (green), SPC (purple), and nuclei (blue) and visualized using IF microscopy (*n* = 3). Original magnification = 40 ×; scale bars = 45 μm. **F** The cells were transfected with 0.3 μg T1α-Luc and 0.1 μg Lac Z plasmids for 24 h, and were then treated with MPT0E028 (0.3 μM) for 0, 8, 12, and 24 h. Luciferase activity was measured. Data are expressed as means ± SEMs from five independent experiments. *p < 0.05, compared with the control group. **G** Primary human AT2 cells were treated with 0.3 μM MPT0E028 for 8, 12, 24, and 48 h, followed by immunoblotting with antibodies specific for T1α, AQP5, SPC, and α-tubulin. **H**–**J** Quantitative analysis of T1α, AQP5, and SPC expression was conducted. Data are expressed as means ± SEMs from six independent experiments. *p < 0.05, compared with the non-treated group. *AQP5* aquaporin 5, *SPC* surfactant protein C.

The alveoli are composed primarily of AT1 cells and of a smaller proportion of AT2 cells [8]. A study demonstrated that in patients with IPF, AT2 cells remain in a transitional state rather than effectively differentiating into AT1 cells [9]. Evidence in the literature suggests that stimulating AT2-to-AT1 cell differentiation through Notch signaling inhibition can ameliorate BLM-induced lung fibrosis in mice [29]. Given that in the current study, MPT0E028 effectively attenuated fibrosis and improved lung function in the therapeutic model of BLM-induced pulmonary fibrosis (Figs. 1, 2 and 3), we hypothesized that its therapeutic effects might be partly mediated by the differentiation of AT2 to AT1 cells. To test this hypothesis, we examined the expression of AT1 cell markers (T1α and AQP5), an AT2 cell marker (SPC), and an alveolar epithelial transitional cell marker (KRT8) in the lung tissues obtained from the therapeutic model of BLM-induced pulmonary fibrosis. IHC analysis revealed that BLM treatment led to an increase in SPC and KRT8 expression and a decrease in T1α and AQP5 levels. However, post-treatment with MPT0E028 (25–100 mg/kg) following BLM treatment reversed these effects in a dose-dependent manner, increasing T1α and AQP5 expression while reducing SPC and KRT8 levels (Fig. 4). To investigate whether pan-HDAC inhibitor contributes to this differentiation process, we assessed the effects of SAHA, a representative pan-HDAC inhibitor, on the therapeutic model of BLM-induced pulmonary fibrosis. Similar to MPT0E028 treatment, SAHA (100 mg/kg) treatment enhanced the expression of T1α and AQP5 while reducing SPC levels in lung tissues (Supplementary Fig. 1 A). We further investigated whether MPT0E028 could induce AT2-to-AT1 cell differentiation in vitro by using MLE-12 cells. MPT0E028 (0.3 μM) treatment induced a time-dependent increase in T1α and AQP5 expression, with expression beginning to increase at 8 h and continuing to rise until 48 h. Correspondingly, SPC expression decreased over the same period (Fig. 5A–D). These findings were further validated through IF staining, which confirmed an increase in T1α expression and a decrease in SPC expression following 24 h MPT0E028 (0.3 μM) treatment in MLE-12 cells (Fig. 5E). In addition, MPT0E028 (0.3 μΜ) induced T1α-luciferase activity in a time-dependent manner in MLE-12 cells (Fig. 5F). In similar to MLE-12 cells, MPT0E028 also induced a time-dependent increase in T1α and AQP5 expression and a decrease in SPC expression over the same period in primary human AT2 cells (Fig. 5G–J). Moreover, SAHA increased T1α expression and reduced SPC expression in MLE-12 cells in a time-dependent manner (Supplementary Fig. 2A–C). Taken together, these findings indicate that both MPT0E028 and SAHA promote AT2-to-AT1 cell differentiation. Notably, the ability of MPT0E028 to restore AT2-to-AT1 differentiation may have contributed to its antifibrotic effects in the BLM-induced pulmonary fibrosis model.

### FoxO1 mediated MPT0E028-induced T1α expression in MLE-12 cells

**Fig. 6:**
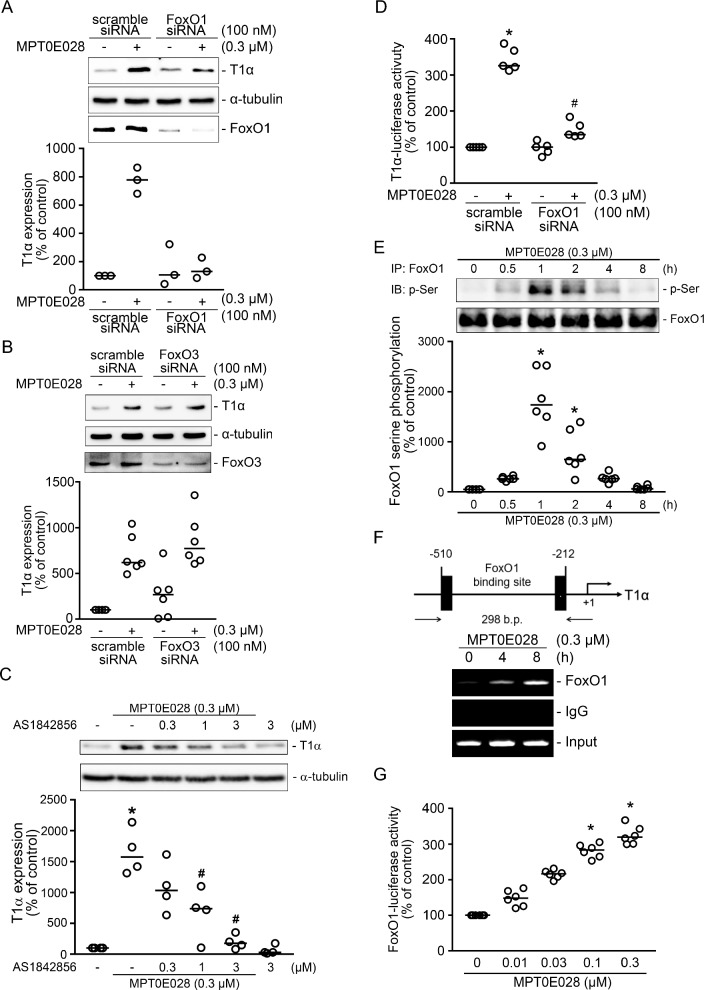
MPT0E028 enhanced FoxO1 recruitment to the T1α promoter and FoxO1-luciferase activity to induce T1α expression in MLE-12 cells. **A** MLE-12 cells were transfected with 100 nM scrambled or FoxO1 siRNA for 24 h, followed by treatment with 0.3 μM MPT0E028 for 24 h. Protein expression levels of T1α, α-tubulin, and FoxO1 were analyzed through immunoblotting with specific antibodies. Data are expressed as means ± SEMs from three independent experiments; *p < 0.05, compared with the scrambled siRNA-treated group. ^#^*p* < 0.05, compared with the group co-treated with MPT0E028 and scrambled siRNA. **B** The cells were transfected with 100 nM scrambled or FoxO3 siRNA for 24 h, followed by treatment with 0.3 μM MPT0E028 for 24 h. Protein expression levels of T1α, α-tubulin, and FoxO3 were analyzed through immunoblotting with specific antibodies. Data are expressed as means ± SEMs from six independent experiments. **C** MLE-12 cells were pretreated with AS1842856 (FoxO1 inhibitor; 0.3, 1, and 3 μM) for 30 min and then treated with 0.3 μM MPT0E028 for 24 h. Protein expression levels of T1α and α-tubulin were analyzed through Western blotting. Data are presented as means ± SEMs from four independent experiments. *p < 0.05, compared with the non-treated group. ^#^*p* < 0.05, compared with the MPT0E028-treated group. **D** The cells were transfected with 100 nM scrambled or FoxO1 siRNA, 0.3 μg T1α-Luc and 0.1 μg Lac Z plasmids for 24 h, and were then treated with MPT0E028 (0.3 μM) for an additional 24 h. Luciferase activity was measured. Data are expressed as means ± SEMs from five independent experiments. *p < 0.05, compared with the control group. ^#^*p* < 0.05, compared with the group co-treated with MPT0E028 and scrambled siRNA. **E** The cells were treated with 0.3 μM MPT0E028 for 0, 0.5, 1, 2, 4, and 8 h. Cells were subsequently subjected to immunoprecipitation using FoxO1-specific antibodies, followed by immunoblot analysis with anti-phospho-serine (p-Ser) or FoxO1-specific antibodies. Equal loading was confirmed by comparable FoxO1 immunoreactivity in each lane. Data are presented as means ± SEMs from six independent experiments. *p < 0.05, compared with the non-treated group. **F** Schematic of the 297-bp ChIP primer located on the T1α promoter. The cells were treated with 0.3 μM MPT0E028 for 0, 4, and 8 h, after which the ChIP assay was performed to detect FoxO1 recruitment on the T1α promoter. Nonimmune IgG served as a negative control, and the input DNA indicated equal chromatin loading (*n* = 3 per condition). **(G)** The cells were transfected with 0.3 μg FoxO1-Luc and 0.1 μg Lac Z plasmids for 24 h and were then treated with MPT0E028 (0.01, 0.03, 0.1, and 0.3 μM) for an additional 24 h. Luciferase activity was measured. Data are expressed as means ± SEMs from six independent experiments. *p < 0.05, compared with the control group. *E028* MPT0E028, *FoxO1* forkhead box O1, *p-Ser* phosphorylated serine.

Research has indicated that FoxO1 activation plays a role in differentiation of AT2 cells to AT1 cells by enhancing binding with Nkx2.1, leading to the suppression of SPC expression in AT2 cells [26]. On the basis of this observation, we hypothesized that MPT0E028 enhances FoxO1 or FoxO3 transcriptional activity, thereby directly upregulating T1α expression and facilitating AT2-to-AT1 differentiation. To determine whether FoxO1 or FoxO3 is required for MPT0E028-induced T1α expression, this study used FoxO1 siRNA and FoxO3 siRNA. As presented in Fig. 6A, B, transfection with FoxO1 siRNA (100 nM) (Fig. 6A) markedly reduced MPT0E028-induced T1α expression, while FoxO3 siRNA (100 nM) had no effect (Fig. 6B) in MLE-12 cells. Furthermore, we also found that AS1842856, an FoxO1 selective inhibitor, inhibited MPT0E028-induced T1α expression in a concentration-dependent manner (Fig. 6C). Moreover, MPT0E028 induced T1α-luciferase activity were markedly reduced by transfection with FoxO1 siRNA (100 nM) in MLE-12 cells (Fig. 6D). To determine whether MPT0E028 induces FoxO1 phosphorylation in MLE-12 cells, we examined phosphorylation levels following MPT0E028 treatment. MPT0E028 (0.3 μM) treatment increased FoxO1 serine phosphorylation, with peak phosphorylation observed at 60 min post-treatment (Fig. 6E). Moreover, a ChIP assay revealed that MPT0E028 (0.3 μΜ) treatment increased FoxO1 recruitment to the FoxO1 binding site on the T1α promoter (Fig. 6F). Furthermore, MPT0E028 (0.01–0.3 μΜ) treatment increased FoxO1-luciferase activity in MLE-12 cells (Fig. 6G). Taken together, these results suggest that FoxO1, but not FoxO3, is involved in MPT0E028-induced T1α expression in MLE-12 cells.

### AMPK activation mediates MPT0E028-induced T1α expression

**Fig. 7:**
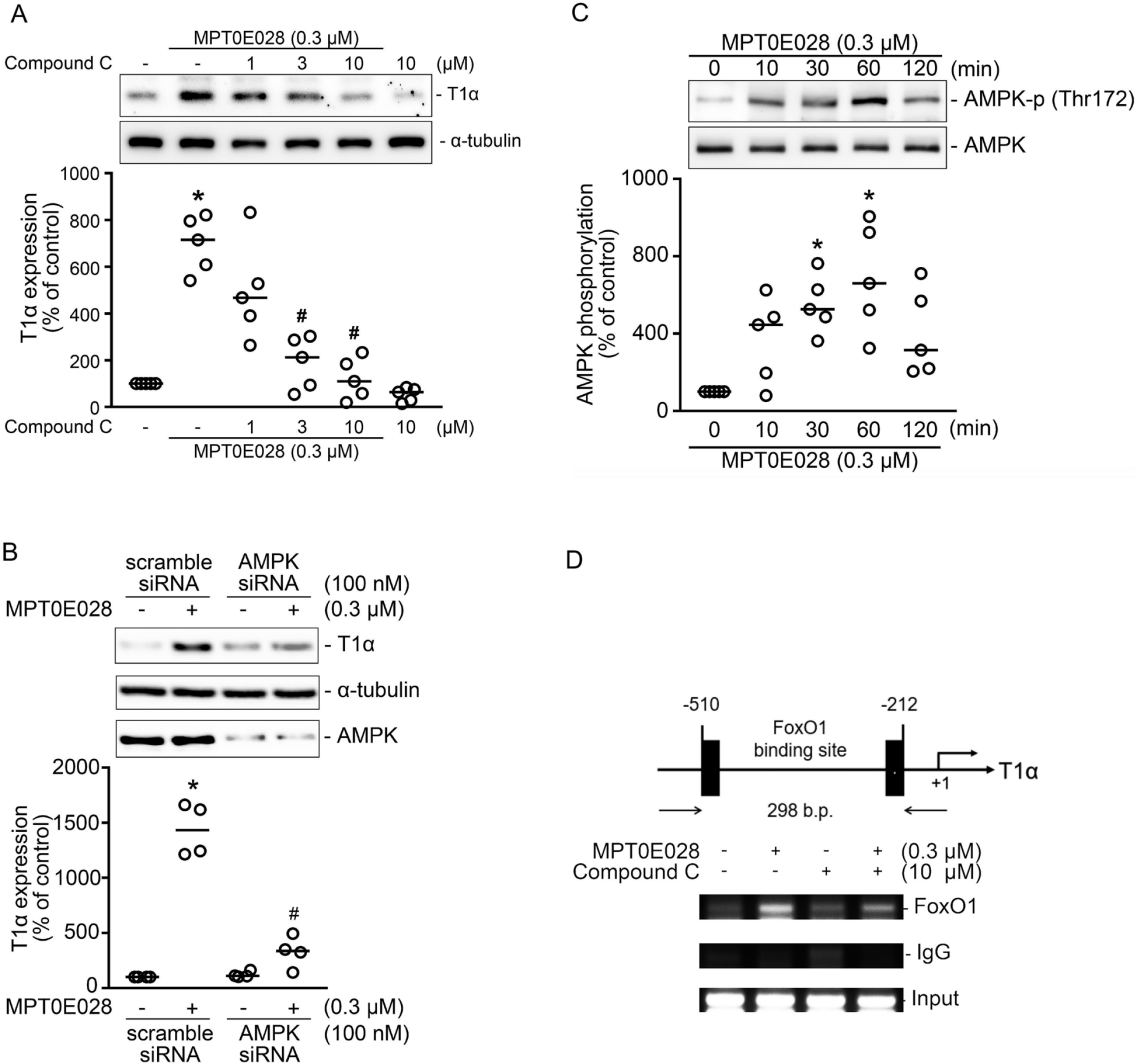
MPT0E028 activated AMPK to enhance FoxO1 recruitment to the T1α promoter and induce T1α expression in MLE-12 cells. **A** MLE-12 cells were pretreated with compound C (AMPK inhibitor; 1, 3, and 10 μM) for 30 min and then treated with 0.3 μM MPT0E028 for 24 h. Protein expression levels of T1α and α-tubulin were analyzed through Western blotting. Data are presented as means ± SEMs from five independent experiments. *p < 0.05, compared with the non-treated group. ^#^*p* < 0.05, compared with the MPT0E028-treated group. **B** The cells were transfected with 100 nM scrambled or AMPK siRNA for 24 h, followed by treatment with 0.3 μM MPT0E028 for 24 h. Protein expression levels of T1α, α-tubulin, and AMPK were analyzed through immunoblotting with specific antibodies. Data are expressed as means ± SEMs from four independent experiments. *p < 0.05, compared with the scrambled siRNA-treated group. ^#^*p* < 0.05, compared with the group co-treated with MPT0E028 and scrambled siRNA. **C** The cells were treated with 0.3 μM MPT0E028 for 10, 30, 60, and 120 min, and p-AMPK (Thr172) and AMPK were detected through immunoblotting with specific antibodies. Data are presented as means ± SEMs from five independent experiments. *p < 0.05, compared with the non-treated group. **D** Schematic of the 297-bp ChIP primer located on the T1α promoter. The cells were pretreated with 10 μM compound C for 30 min and then stimulated with 0.3 μM MPT0E028 for 8 h. A ChIP assay was subsequently performed to evaluate FoxO1 recruitment at the T1α promoter. Nonimmune IgG served as a negative control, whereas input DNA confirmed equal chromatin loading (*n* = 3 per condition). *AMPK* AMP-activated protein kinase, *p-AMPK* phosphorylated AMPK, *ChIP* chromatin immunoprecipitation.

AMPK activation was demonstrated to promote the differentiation of AT2 cells to AT1 cells by increasing energy metabolism in primary murine AT2 cells [24]. Additionally, such activation was demonstrated to increase FoxO1 transcriptional activity, facilitating oxygen-regulated protein 150 (ORP150) expression to alleviate cigarette smoke extract-induced endoplasmic reticulum stress in human primary bronchial epithelial cells [25]. Therefore, we investigated whether AMPK activation mediated MPT0E028-induced T1α expression. We treated MLE-12 cells with compound C, an AMPK inhibitor, and the results revealed that compound C inhibited MPT0E028-induced T1α expression in a concentration-dependent manner (Fig. 7A). Furthermore, transfection with AMPK siRNA (100 nM) markedly reduced MPT0E028-induced T1α expression in MLE-12 cells (Fig. 7B). Subsequently, we investigated whether MPT0E028 (0.3 μΜ) activates AMPK by assessing AMPK phosphorylation at Thr172 through an immunoblotting analysis [30]. As indicated in Fig. 7C, MPT0E028 (0.3 μΜ) increased AMPK phosphorylation in a time-dependent manner, with phosphorylation detectable at 10 min and peaking at 60 min. These findings suggest that AMPK activation is involved in the regulation of T1α expression by MPT0E028. Furthermore, a ChIP assay revealed that compound C inhibited MPT0E028-increased FoxO1 recruitment to the T1α promoter (Fig. 7D). These findings indicate that MPT0E028 promotes AMPK activation, which in turn facilitates FoxO1 recruitment to the T1α promoter, leading to increased T1α expression in MLE-12 cells.

### MPT0E028 enhanced ATM acetylation and activation to induce T1α expression in MLE-12 cells

**Fig. 8:**
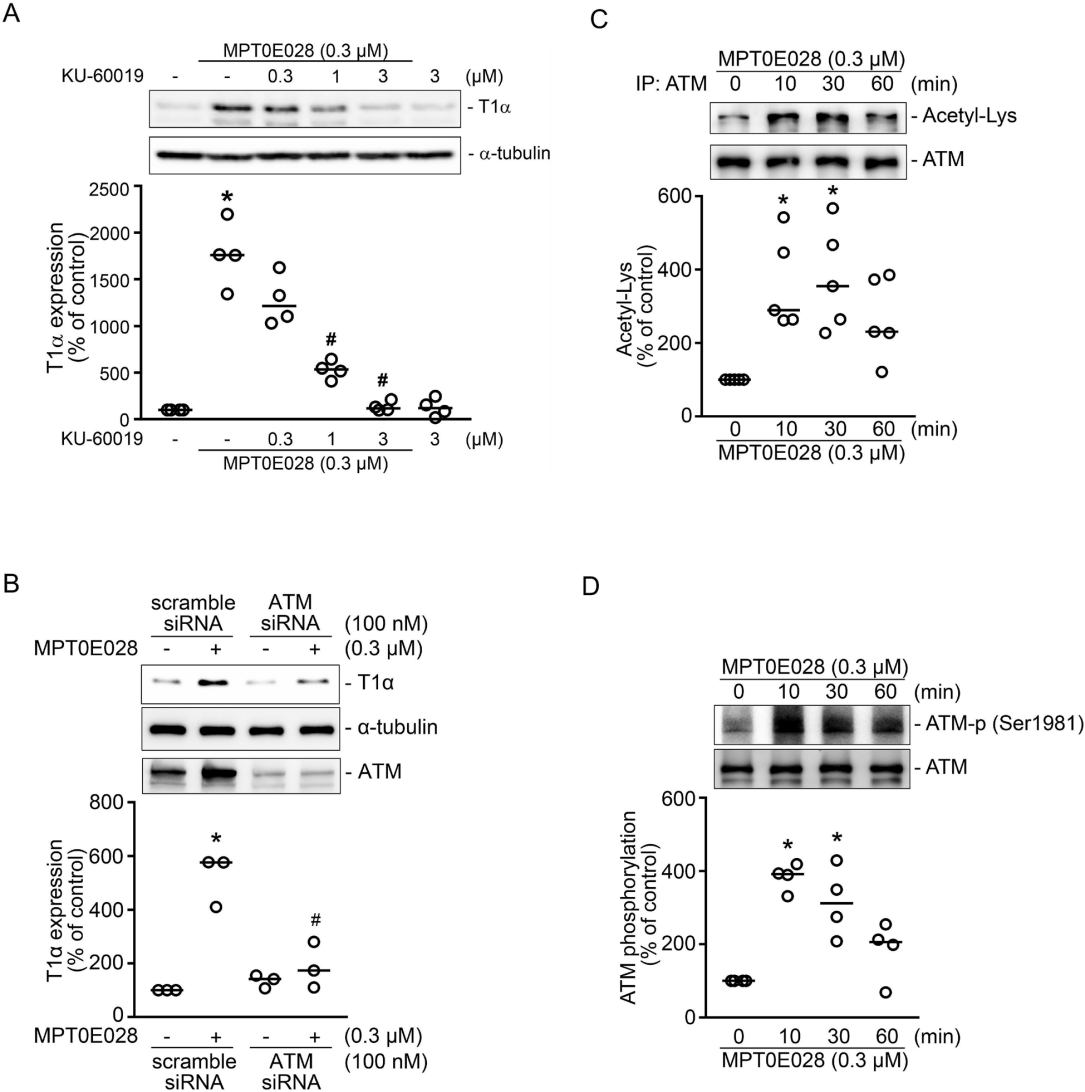
ATM acetylation and phosphorylation were involved in MPT0E028-indcued T1α expression in MLE-12 cells. **A** MLE-12 cells were pretreated with KU-60019 (ATM inhibitor; 0.3, 1, and 3 μM) for 30 min, and then treated with 0.3 μM MPT0E028 for 24 h. Protein expression levels of T1α and α-tubulin were analyzed through Western blotting. Data are presented as means ± SEMs from four independent experiments. *p < 0.05, compared with the non-treated group. ^#^*p* < 0.05, compared with the MPT0E028-treated group. **B** MLE-12 cells were transfected with either scrambled or ATM siRNA (100 nM) for 24 h, with this followed by treatment with 0.3 μM MPT0E028 for 24 h. The protein expression levels of T1α, α-tubulin, and ATM were assessed through immunoblotting with specific antibodies. Data are presented as means ± SEMs from three independent experiments. *p < 0.05, compared with the scrambled siRNA-treated group; ^#^*p* < 0.05, compared with the group co-treated with MPT0E028 and scrambled siRNA. **C** The cells were treated with 0.3 μM MPT0E028 for 10, 30, and 60 min and then harvested for IP of ATM. The immunoprecipitated samples were prepared for immunoblotting with antibodies specific for acetylated lysine and ATM. Data are expressed as means ± SEMs from five independent experiments. *p < 0.05, compared with the untreated group. **D** The cells were treated with MPT0E028 (0.3 μM) for 10, 30, and 60 min and then collected for immunoblotting. p-ATM (Ser1981) and ATM were detected using specific antibodies. Data are expressed as means ± SEMs from four independent experiments. *p < 0.05, compared with the untreated group. *Acetyl-Lys* acetylated lysine, *ATM* ataxia-telangiectasia mutated, *p-ATM* phosphorylated ATM.

**Fig. 9:**
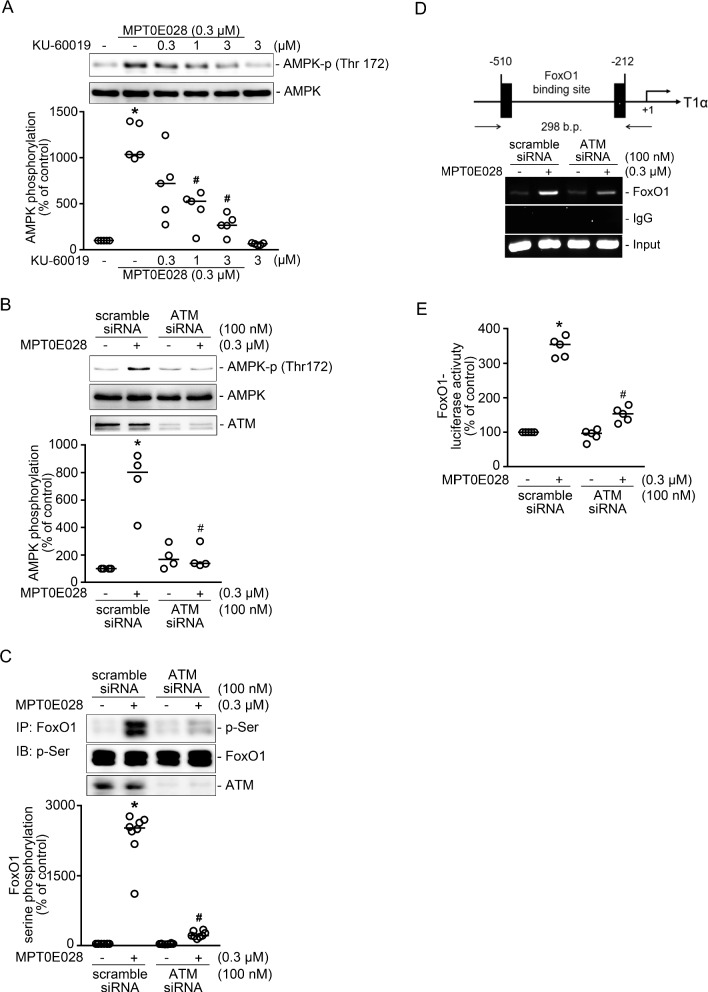
ATM mediated AMPK/FoxO1 signaling by MPT0E028 stimulation in MLE-12 cells. **A** MLE-12 cells were pretreated with KU-60019 (ATM inhibitor; 0.3, 1, and 3 μM) for 30 min, and then treated with 0.3 μM MPT0E028 for 30 min. p-AMPK (Thr172) and AMPK were detected through immunoblotting with specific antibodies. Data are presented as means ± SEMs from five independent experiments. *p < 0.05, compared with the non-treated group. ^#^*p* < 0.05, compared with the MPT0E028-treated group. **B** The cells were transfected with scrambled or ATM siRNA (100 nM). After 24 h, the cells were incubated with 0.3 μM MPT0E028 for 30 min. p-AMPK (Thr172) and AMPK were detected through immunoblotting with specific antibodies. Data are presented as means ± SEMs from four independent experiments. *p < 0.05, compared with the non-treated group with scrambled siRNA. ^#^*p* < 0.05, compared with the MPT0E028-treated group with scrambled siRNA. **C** The cells were transfected with scrambled or ATM siRNA (100 nM). After 24 h, the cells were incubated with 0.3 μM MPT0E028 for 60 min. Cells were subsequently subjected to immunoprecipitation using FoxO1-specific antibodies, followed by immunoblot analysis with anti-phospho-serine (p-Ser) or FoxO1-specific antibodies. Equal loading was confirmed by comparable FoxO1 immunoreactivity in each lane. Data are presented as means ± SEMs from eight independent experiments. *p < 0.05, compared with the non-treated group. **D** Schematic of the 297-bp ChIP primer located on the T1α promoter. The cells were transfected with scrambled or ATM siRNA (100 nM) for 24 h and then treated with 0.3 μM MPT0E028 for 8 h, with this followed by ChIP assay. Nonimmune IgG served as a negative control, whereas input DNA confirmed equal chromatin loading (*n* = 3 per condition). **E** The cells were transfected with 100 nM scrambled or ATM siRNA, 0.3 μg FoxO1-Luc and 0.1 μg Lac Z plasmids for 24 h, and were then treated with MPT0E028 (0.3 μM) for an additional 24 h. Luciferase activity was measured. Data are expressed as means ± SEMs from five independent experiments. *p < 0.05, compared with the control group. ^#^*p* < 0.05, compared with the group co-treated with MPT0E028 and scrambled siRNA. *AMPK* AMP-activated protein kinase; *p-AMPK* phosphorylated AMPK, *ATM* ataxia-telangiectasia mutated, *ChIP* chromatin immunoprecipitation, *FoxO1* forkhead box O1, *IP* immunoprecipitation, *p-Ser* phosphorylated serine

ATM is essential for the rapid regeneration of proximal airway epithelium following influenza A virus infection [31]. Deletion of ATM has been shown to exacerbate bleomycin-induced pulmonary fibrosis in mice and reduce AT2 cell viability [22]. To investigate whether ATM is involved in MPT0E028-stimulated T1α expression, we treated ATM inhibitor (KU-60019) and transfected with ATM siRNA in MLE-12 cells. As indicated in Fig. 8A, treated MLE-12 cells with KU-60019, an ATM inhibitor, attenuated MPT0E028-stimulated T1α expression in a concentration-dependent manner. Additionally, transfection with ATM siRNA significantly reduced MPT0E028-induced T1α expression (Fig. 8B). Acetylation of ATM is essential for its autophosphorylation, and phosphorylation at serine 1981 is widely recognized as a hallmark of ATM activation [19, 32]. To determine whether MPT0E028 promotes ATM acetylation in MLE-12 cells, we examined acetylation levels following MPT0E028 treatment. The results revealed that MPT0E028 (0.3 μM) treatment significantly increased ATM acetylation, with peak acetylation observed at 30 min post-treatment (Fig. 8C). To confirm whether MPT0E028-induced ATM acetylation led to ATM activation, we further assessed ATM phosphorylation at serine 1981 and revealed that MPT0E028 (0.3 μΜ) treatment significantly increased ATM phosphorylation, with the maximum effect observed at 30 min post-treatment (Fig. 8D). We also explored whether ATM activation participated in the MPT0E028-activated AMPK/FoxO1 signaling pathway. As indicated in Fig. 9A, treated MLE-12 cells with KU-60019, an ATM inhibitor, suppressed MPT0E028-mediated AMPK phosphorylation in a concentration-dependent manner. Similarly, transfection with ATM siRNA (100 nM) suppressed MPT0E028-induced AMPK phosphorylation (Fig. 9B). Furthermore, transfection with ATM siRNA (100 nM) markedly reduced MPT0E028-induced FoxO1 serine phosphorylation in MLE-12 cells (Fig. 9C). Additionally, ATM siRNA (100 nM) transfection reduced the MPT0E028-induced recruitment of FoxO1 to the T1α promoter region (Fig. 9D). MPT0E028 (0.3 μΜ)-induced FoxO1-luciferase activity was markedly reduced by transfection with ATM siRNA (100 nM) (Fig. 9E). These results suggest that MPT0E028 promotes ATM acetylation, leading to its activation, which subsequently triggers the AMPK/FoxO1 signaling pathway to induce T1α expression in MLE-12 cells.

### MPT0E028 enhanced FoxO1 phosphorylation in AT2 cells in a therapeutic model of BLM-induced pulmonary fibrosis in mice

**Fig. 10:**
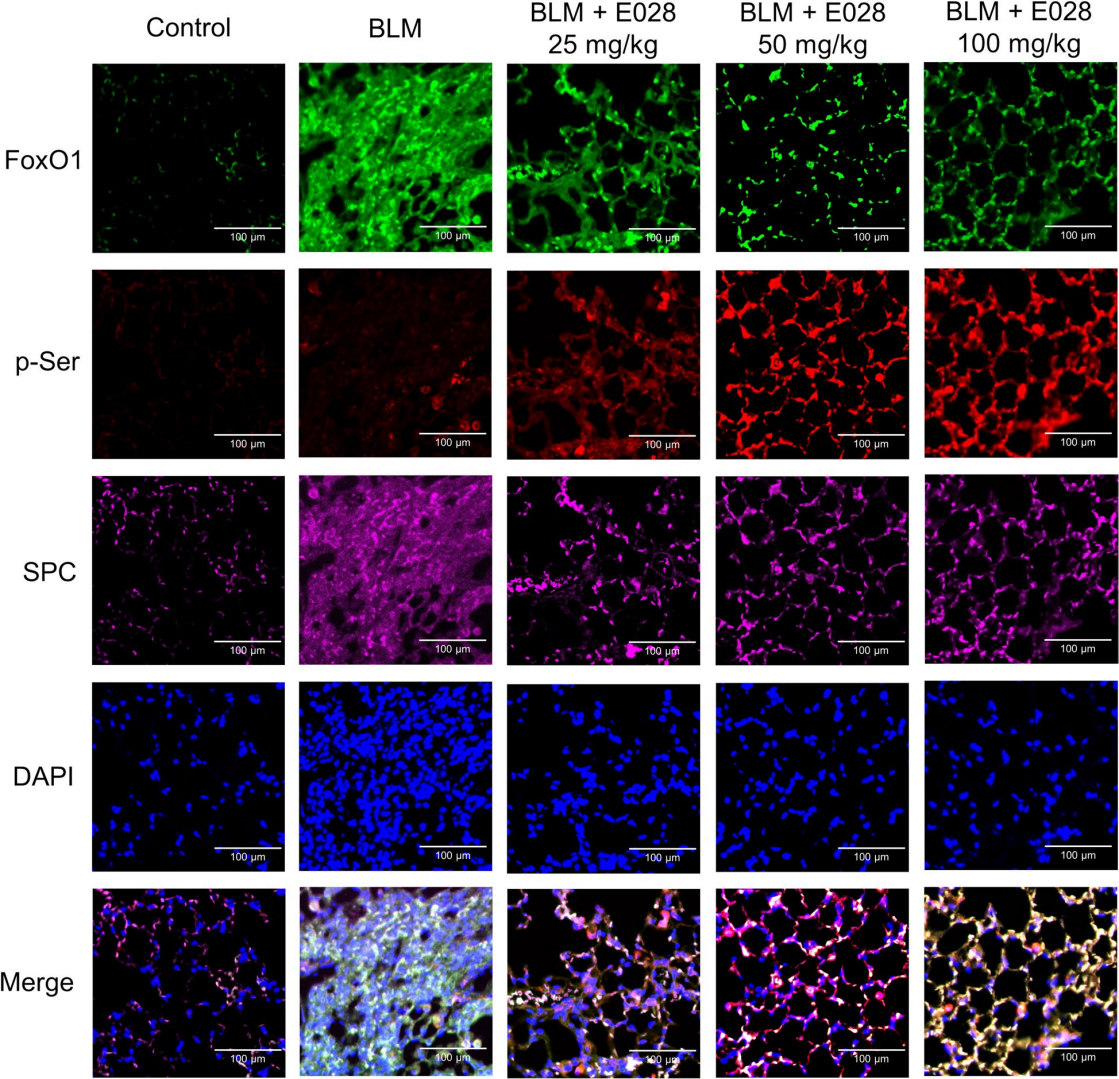
MPT0E028 enhanced FoxO1 phosphorylation in AT2 cells in a therapeutic model of BLM-induced pulmonary fibrosis in mice. On day 32, whole lungs were harvested from mice treated with PBS, BLM, or BLM + MPT0E028 (25, 50, and 100 mg/kg). Whole lungs were obtained from the mice and fixed with formaldehyde for 24 h, embedded in paraffin, and subjected to IF staining for FoxO1, p-Ser, and SPC. Nuclei were counterstained with DAPI. Representative IF images show FoxO1 (green), p-Ser (red), SPC (purple), DAPI (blue), and merged signals. Original magnification = 20 ×; scale bars = 100 μm. n = 6 mice per group. *BLM* bleomycin, *E028* MPT0E028, *IF* immunofluorescence, *p-Ser* phosphorylated serine, *SPC* surfactant protein C

In this study, we found that MPT0E028 induced T1α expression in AT2 cells through the phosphorylation of FoxO1. To further determine whether the MPT0E028-indcued FoxO1 phosphorylation in AT2 cell also occurs in a therapeutic model of BLM-induced pulmonary fibrosis in mice, we performed immunofluorescence staining of mouse lung sections for FoxO1 (green), p-Ser (red), and the AT2 cell marker SPC (purple). IF analysis revealed that BLM treatment increased FoxO1 expression in the lung tissues of mice, whereas subsequent treatment with MPT0E028 (25–100 mg/kg) reduced FoxO1 expression. Further analysis showed that FoxO1 was colocalized with p-Ser in SPC-positive AT2 cells. Although BLM treatment increased FoxO1 expression, it did not enhance FoxO1 serine phosphorylation in AT2 cells. In contrast, subsequent treatment with MPT0E028 (25–100 mg/kg) dose-dependently enhanced FoxO1 serine phosphorylation in AT2 cells (Fig. 10). These results suggest that MPT0E028 promotes FoxO1 serine phosphorylation in AT2 cells in a therapeutic model of BLM-induced pulmonary fibrosis in mice.

## Discussion

This study demonstrated that treatment with MPT0E028, a novel pan-HDAC inhibitor, significantly improved fibrosis scores, reduced the expression of fibrogenic proteins, and mitigated lung function decline in a therapeutic model of BLM-induced pulmonary fibrosis in mice. Furthermore, in a therapeutic model of BLM-induced pulmonary fibrosis in mice, treatment with MPT0E028 led to an increase in AT1 cell markers, including T1α and AQP5, and a reduction in the expression of the AT2 marker SPC and an alveolar epithelial transitional cell marker KRT8. In AT2 cells (MLE-12 cells and primary human AT2 cells), MPT0E028 induced T1α and AQP5 expression and reduced SPC expression in a time-dependent manner. Moreover, MPT0E028 promoted ATM acetylation and activation, leading to the phosphorylation of AMPK in MLE-12 cells. This activation subsequently stimulated FoxO1, which in turn enhanced T1α expression in MLE-12 cells. Importantly, we have demonstrated that MPT0E028 is the first pan-HDAC inhibitor shown to activate ATM acetylation-mediated AMPK/FoxO1 signaling to induce AT2-to-AT1 differentiation in a therapeutic model of BLM-induced pulmonary fibrosis in mice. Taken together, these findings indicate that MPT0E028 improves lung function by ameliorating the therapeutic model of BLM-induced pulmonary fibrosis, potentially through the suppression of fibrogenic protein expression and the promotion of AT2-to-AT1 cell differentiation.

Our findings reveal that post-treatment with MPT0E028, initiated on day 10 following BLM administration, significantly reduced BLM-induced expression of fibrogenic proteins and increase in fibrosis scores, suggesting that suppression of fibrogenic protein expression contributes to the improvement of fibrotic injury. This finding is consistent with the results of our previous study, which demonstrated that pretreatment with MPT0E028 prevents BLM-induced pulmonary fibrosis by downregulating CTGF expression, thereby reducing fibronectin, collagen I, and α-SMA levels [13]. Furthermore, postadministration of MPT0E028 improved BLM-induced impairments in lung function in mice, as evidenced by increased lung compliance and FEV_0.1_, along with reduced lung elastance and airway resistance. Collectively, these findings suggest that the enhancement of lung function following MPT0E028 treatment is partially attributable to its antifibrotic effects in BLM-induced pulmonary fibrosis. Notably, our findings are consistent with those of a study indicating that the HDAC inhibitor SAHA ameliorates pulmonary fibrosis and enhances lung function [16]. Further evaluation in chronic fibrosis model, as well as in TGF-β-induced fibrosis model will be important to define the therapeutic potential of MPT0E028. In addition, previous studies have demonstrated that oral pirfenidone (200 mg/kg) and nintedanib (60 mg/kg) effectively attenuate bleomycin-induced pulmonary fibrosis, reduce fibrogenic protein expression, and improve lung function [27, 28]. In this study, we found that high-dose MPT0E028 (100 mg/kg) exhibited a comparable efficacy to pirfenidone (200 mg/kg) and nintedanib (60 mg/kg) in reducing pulmonary fibrosis and improving lung function in the therapeutic model of BLM-induced pulmonary fibrosis.

Re-epithelialization of damaged alveoli, including differentiation of AT2 cells into AT1 cells, is critical in the resolution of pulmonary fibrosis [5]. T1α is one of the most established and functionally relevant AT1 cell markers, and its expression is associated with AT2-to-AT1 cell differentiation [33]. T1α-deficient mice were reported to exhibit significantly reduced numbers of AT1 cells and decreased expression of the AT1 cell marker Aqp5 mRNA, with this leading to abnormal lung architecture and impaired air–blood barrier formation [33]. Furthermore, T1α^+^ AT1 cells were reported to primarily originate from SPC^+^ AT2 cells during repair and re-epithelialization in mouse models of lung injury induced by lipopolysaccharide and hydroxy chloride [34]. KRT8 has been identified as a marker of alveolar differentiation intermediate (ADI) population, which accumulate during aberrant repair and have been implicated in fibrotic progression [35]. In the present study, we observed an increase in KRT8 expression in the BLM group, and treatment with MPT0E028 reduced the KRT8 expression. This reduction was accompanied by increased expression of T1α and AQP5 and decreased expression of SPC. These results suggest that MPT0E028 does not cause KRT8^+^ alveolar epithelial transitional cells retention; instead, it promotes their progression toward differentiation into mature AT1 cells. Additionally, in MLE-12 cells and primary human AT2 cells, MPT0E028 induced the differentiation of AT2 cells to AT1 cells. Furthermore, MPT0E028 induced T1α-luciferase activity in MLE-12 cells. These results suggest that MPT0E028 ameliorates BLM-induced pulmonary fibrosis by promoting AT2-to-AT1 cell differentiation, thereby facilitating re-epithelialization in the fibrotic injury. Our findings are consistent with those of another study demonstrating that SAHA, a pan-HDAC inhibitor, has the potential to induce AT2-to-AT1 cell differentiation [17].

Our findings reveal that FoxO1 siRNA, but not FoxO3 siRNA, inhibited MPT0E028-induced T1α expression in MLE-12 cells. Additionally, FoxO1 selective inhibitor (AS1842856) attenuated MPT0E028-induced T1α expression, and FoxO1 siRNA inhibited MPT0E028-induced T1α-luciferase activity. Furthermore, MPT0E028 enhanced FoxO1 serine phosphorylation, FoxO1 recruitment to the T1α promoter, and FoxO1-luciferase activity. These results indicated that MPT0E028 mediated T1α expression through FoxO1 phosphorylation in AT2 cells. We also found that MPT0E028 promotes FoxO1 serine phosphorylation in AT2 cells in a therapeutic model of BLM-induced pulmonary fibrosis in mice. In consistent with the results from MLE-12 cells, these results further indicate that FoxO1 phosphorylation plays a key role in AT2-to-AT1 cell differentiation. In addition, previous study has demonstrated that FoxO1 contributes to AT2-to-AT1 cell differentiation by interacting with Nkx2.1 and disrupting Nkx2.1 binding to the SPC promoter, thereby reducing SPC expression in MLE-15 cells [26]. In the current study, we also found that BLM-induced FoxO1 expression in the lung tissues of BLM-induced pulmonary fibrosis in mice was reduced by MPT0E028 treatment. Previous study revealed that BLM stimulation increased FoxO1 expression in the lung tissue of BLM-induced lung fibrosis model [36]. However, the mechanism by which MPT0E028 regulates FoxO1 expression requires further investigation.

In the current study, we noted that compound C, an AMPK inhibitor, inhibited MPT0E028-stimulated T1α expression and the recruitment of FoxO1 to the T1α promoter. AMPK siRNA knockdown similarly attenuated MPT0E028-induced T1α expression. Furthermore, MPT0E028 induced AMPK phosphorylation in a time-dependent manner. These results suggest that AMPK-mediated FoxO1 activation is involved in MPT0E028-induced T1α expression. These results are consistent with those of previous research demonstrating that AMPK is required for AT2-to-AT1 cell differentiation, which facilitated alveolar regeneration in a BLM-induced pulmonary fibrosis mouse model [24]. Previous study revealed that AMPK enhances FoxO1 transcriptional activity, leading to the upregulation of manganese superoxide dismutase and catalase, which contribute to the antioxidant effects of resveratrol [37]. Furthermore, metformin, an AMPK activator, has been reported to activate AMPK and promote alveolar epithelial regeneration, including enhanced AT2 proliferation and differentiation [38]. Another study has demonstrated that AMPK activation by metformin can increase FoxO1 activity in endometrial cancer cells [39].

HDAC inhibitors have been demonstrated to not only increase the acetylation of histone protein, thereby regulating gene expression, but also modulate the acetylation levels of nonhistone proteins, affecting their stability, protein–protein interactions, and enzymatic activity. These modifications contribute to the activation or inactivation of signaling pathways and various biological processes [40, 41]. For example, a study indicated that valproic acid (VPA), a pan-HDAC inhibitor, increases heat shock protein 70 acetylation, leading to cell cycle arrest and apoptosis in human epidermal growth factor receptor 2-expressing breast cancer cells [42]. Similarly, our previous study demonstrated that MPT0E028 enhances MKP-1 acetylation and activation, thereby suppressing CTGF expression in human lung fibroblasts [13]. In the present study, MPT0E028 increased ATM acetylation and phosphorylation in MLE-12 cells. Both ATM selective inhibitor (KU-60019) and ATM siRNA transfection suppressed MPT0E028-induced T1α expression. ATM knockdown reduced MPT0E028-induced AMPK phosphorylation, FoxO1 serine phosphorylation, FoxO1 recruitment to the T1α promoter, and FoxO1-luciferase activity in MLE-12 cells. These findings indicate that MPT0E028 promotes ATM activation by enhancing ATM acetylation, thereby stimulating AMPK/FoxO1 signaling to upregulate T1α expression in AT2 cells. These findings are consistent with previous findings indicating that TSA promotes ATM acetylation, leading to increased ATM phosphorylation at serine 1981 and activation of the ATM-dependent DNA damage signaling pathway [20]. Another study demonstrated that VPA, a pan-HDAC inhibitor, activates the ATM/AMPK/Unc-51 like autophagy activating kinase 1 signaling pathway, thereby enhancing niraparib-induced cellular death in ovarian and breast cancer cells [43].

The present study demonstrated that MPT0E028 promotes differentiation of AT2 cells to AT1 cells, thereby facilitating re-epithelialization and contributing to the resolution of BLM-induced pulmonary fibrosis. However, several limitations necessitate further investigation; the resolution of pulmonary fibrosis involves various processes, including the clearance fibroproliferative stimuli, elimination of activated myofibroblasts, and degradation of the ECM [5]. Future research is required to elucidate the precise mechanisms underlying MPT0E028’s antifibrotic effects and to determine its potential therapeutic applications in pulmonary fibrosis.

## Conclusions

**Fig. 11:**
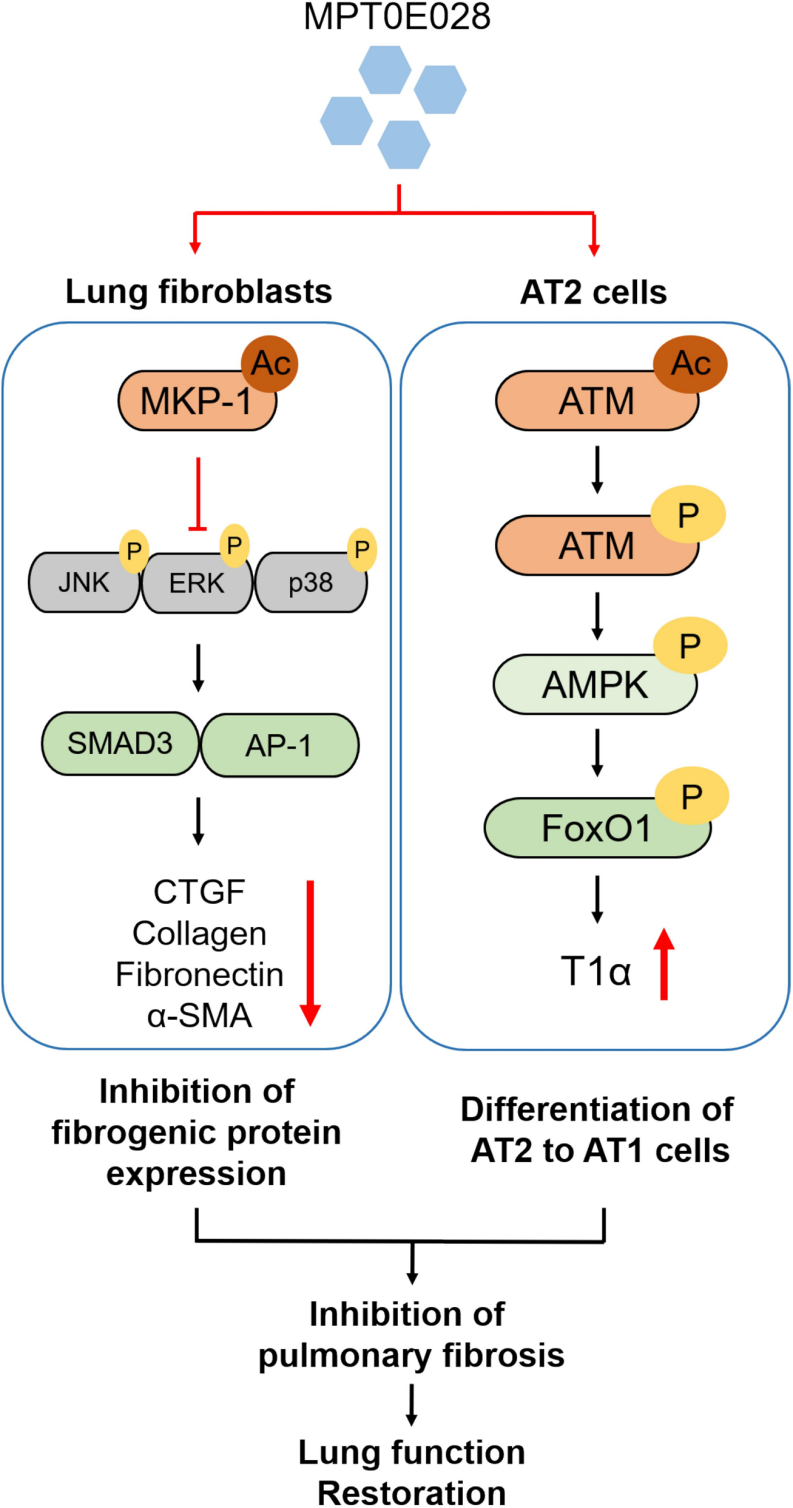
Schematic of MPT0E028-mediated inhibition of BLM-induced pulmonary fibrosis in mice. MPT0E028 effectively mitigated pulmonary fibrosis by reducing the expression of key fibrogenic proteins, including CTGF, collagen, fibronectin, and α-SMA, in a BLM-induced pulmonary fibrosis in mice. In human lung fibroblasts, MPT0E028 suppresses CTGF expression by enhancing MKP-1 acetylation and activity, thereby promoting the dephosphorylation of JNK, p38, and ERK, attenuating SMAD3/AP-1 transcriptional activity, and subsequently reducing downstream fibrogenic protein expression. Additionally, MPT0E028 induced ATM acetylation and activation, triggering AMPK activation. Subsequently, MPT0E028-induced AMPK activation enhanced FoxO1 activity, leading to the upregulation of T1α expression in AT2 cells and facilitating AT2-to-AT1 cell differentiation. Collectively, these effects contributed to MPT0E028 ameliorating pulmonary fibrosis and restoring lung function in a BLM-induced pulmonary fibrosis in mice. *Ac* acetylation, *AT1* type 1 alveolar epithelial cells, *AT2* type 2 alveolar epithelial cells, *AMPK* AMP-activated protein kinase, *ATM* ataxia-telangiectasia mutated, *CTGF* connective tissue growth factor; *α-SMA* α-smooth muscle actin, *FoxO1* forkhead box O1, *P* phosphorylation, *MKP-1* mitogen-activated protein kinase phosphatase-1

This study highlighted the potential of MPT0E028, a pan-HDAC inhibitor, as a therapeutic agent for pulmonary fibrosis. In this study, MPT0E028 exerts its antifibrotic effects by reducing the expression of fibrogenic protein and promoting the differentiation of AT2 cells into AT1 cells through the ATM/AMPK/FoxO1 signaling pathway. Our previous study also demonstrated that MPT0E028 suppresses CTGF expression and downstream fibrogenic protein expression through MKP-1 acetylation in human lung fibroblasts [13]. As illustrated in Fig. 11, these effects collectively contributed to the amelioration of pulmonary fibrosis and restoration of lung function in the BLM-induced pulmonary fibrosis mouse model.

## Supplementary Information

Supplementary Material 1. Fig. 1. SAHA enhanced T1α and AQP5 expression while reducing SPC levels in the lung tissues from a therapeutic model of BLM-induced pulmonary fibrosis in mice. A After 32 days, whole lungs were collected from mice treated with PBS, BLM, or BLM + SAHAand fixed in formaldehyde for 24 h. The lungs were embedded in paraffin and subjected to IHC staining for T1α, AQP5, and SPC. n = 3 per group; original magnification = 20 ×; scale bars = 100 μm. BLM, bleomycin; SAHA, suberoylanilide hydroxamic acid; SPC, surfactant protein C; AQP5, aquaporin 5

Supplementary Material 2. Fig. 2. SAHA upregulated T1α expression while reducing SPC expression in AT2 cells. A MLE-12 cells were treated with 0.3 μM SAHA for 8, 12, 24, and 48 h. Immunoblotting was performed to detect the levels of T1α, SPC, and α-tubulin. B and C Quantitative analysis of T1α and SPC expression was performed after normalization to normalized to α-tubulin, with data presented as means ± SEMs from three independent experiments. *p < 0.05, compared with the non-treated control group. Abbreviations: SAHA, suberoylanilide hydroxamic acid; SPC, surfactant protein C; MLE-12, murine AT2

## Data Availability

No datasets were generated or analysed during the current study.

## Abbreviations

Ac: Acetylation
Acetyl-Lys: Acetylated lysine
ADI: Alveolar differentiation intermediate
α-SMA: α-Smooth muscle actin
AMPK: AMP-activated protein kinase
AT1: Type 1 alveolar epithelial cells
AT2: Type 2 alveolar epithelial cells
ATM: Ataxia-telangiectasia mutated
AQP5: Aquaporin 5
BLM: Bleomycin
BSA: Bovine serum albumin
ChIP: Chromatin immunoprecipitation
CTGF: Connective tissue growth factor
E028: MPT0E028
ECM: Extracellular matrix
ECL: Enhanced chemiluminescence
FBS: Fetal bovine serum
FoxO1: Forkhead box O1
HDAC: Histone deacetylase
HRP: Horseradish peroxidase
IF: Immunofluorescence
IP: Immunoprecipitation
IPF: Idiopathic pulmonary fibrosis
KRT8: Keratin 8
MEM: Minimum essential medium
MKP-1: Mitogen-activated protein kinase phosphatase-1
NEAAs: Nonessential amino acids
PBS: Phosphate-buffered saline
PCR: Polymerase chain reaction
p-AMPK: Phosphorylated AMPK
p-ATM: Phosphorylated ATM
p-Ser: Phosphorylated serine
PVDF: Polyvinylidene difluoride
SAHA: Suberoylanilide hydroxamic acid
SDS-PAGE: Sodium dodecyl sulfate polyacrylamide gel electrophoresis
siRNA: Small interfering RNA
SPC: Surfactant protein C
