# Supplementary figures and images for: MPT0E028, a pan-HDAC inhibitor, ameliorates bleomycin-induced pulmonary fibrosis by promoting AT2-to-AT1 differentiation through the ATM/AMPK/FoxO1 pathway
Source: J Biomed Sci. 2026 Jul 21;33:76. doi: 10.1186/s12929-026-01281-8 (PMC13390252; doi:10.1186/s12929-026-01281-8)

1    **Supplementary Figure 1.**

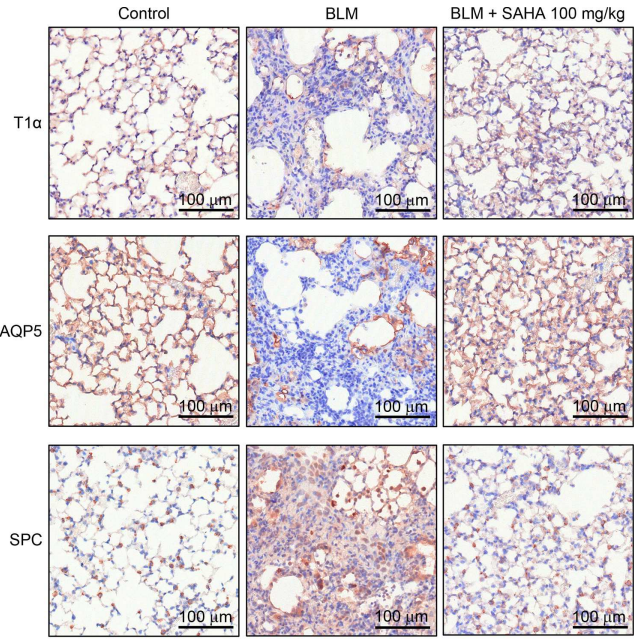

Supplement: Supplementary file 1 — Supplementary Material 1. Fig. 1. SAHA enhanced T1α and AQP5 expression while reducing SPC levels in the lung tissues from a therapeutic model of BLM-induced pulmonary fibrosis in mice. A After 32 days, whole lungs were collected from mice treated with PBS, BLM, or BLM + SAHAand fixed in formaldehyde for 24 h. The lungs were embedded in paraffin and subjected to IHC staining for T1α, AQP5, and SPC. n = 3 per group; original magnification = 20 ×; scale bars = 100 μm. BLM, bleomycin; SAHA, suberoylanilide hydroxamic acid; SPC, surfactant protein C; AQP5, aquaporin 5 [file 12929_2026_1281_MOESM1_ESM.pdf]

1 **Supplementary Figure 2.**

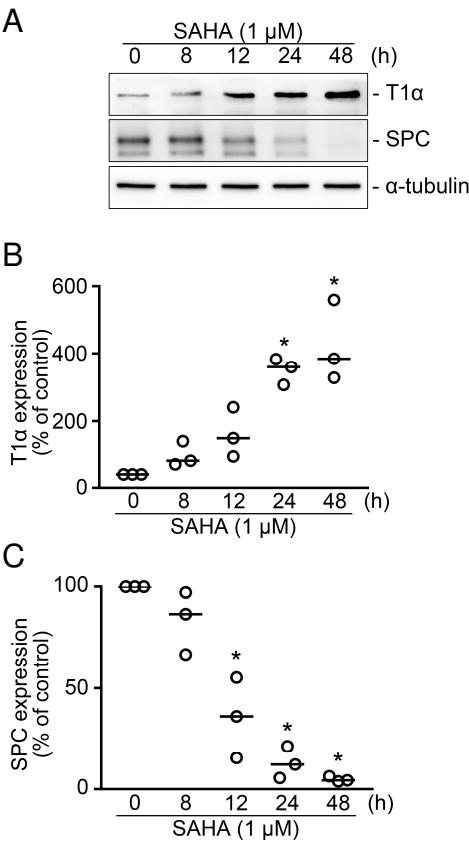

Supplement: Supplementary file 2 — Supplementary Material 2. Fig. 2. SAHA upregulated T1α expression while reducing SPC expression in AT2 cells. A MLE-12 cells were treated with 0.3 μM SAHA for 8, 12, 24, and 48 h. Immunoblotting was performed to detect the levels of T1α, SPC, and α-tubulin. B and C Quantitative analysis of T1α and SPC expression was performed after normalization to normalized to α-tubulin, with data presented as means ± SEMs from three independent experiments. *p < 0.05, compared with the non-treated control group. Abbreviations: SAHA, suberoylanilide hydroxamic acid; SPC, surfactant protein C; MLE-12, murine AT2 [file 12929_2026_1281_MOESM2_ESM.pdf]
